# Summertime tropospheric ozone source apportionment study in the Madrid region (Spain)

**DOI:** 10.5194/acp-24-4949-2024

**Published:** 2023-10-06

**Authors:** David de la Paz, Rafael Borge, Juan Manuel de Andrés, Luis Tovar, Golam Sarwar, Sergey L. Napelenok

**Affiliations:** 1 Laboratory of Environmental Modelling, Department of Chemical & Environmental Engineering, Universidad Politécnica de Madrid, (UPM), c/ José Gutiérrez Abascal 2, 28006 Madrid, Spain; 2 Center for Environmental Measurement & Modeling, U.S. Environmental Protection Agency, Research Triangle Park, NC 27711, USA

## Abstract

The design of emission abatement measures to effectively reduce high ground-level ozone (O_3_) concentrations in urban areas is very complex. In addition to the strongly non-linear chemistry of this secondary pollutant, precursors can be released by a variety of sources in different regions, and locally produced O_3_ is mixed with that transported from the regional or continental scales. All of these processes depend also on the specific meteorological conditions and topography of the study area. Consequently, high-resolution comprehensive modeling tools are needed to understand the drivers of photochemical pollution and to assess the potential of local strategies to reduce adverse impacts from high tropospheric O_3_ levels. In this study, we apply the Integrated Source Apportionment Method (ISAM) implemented in the Community Multiscale Air Quality (CMAQ v5.3.2) model to investigate the origin of summertime O_3_ in the Madrid region (Spain). Consistent with previous studies, our results confirm that O_3_ levels are dominated by non-local contributions, representing around 70 % of mean values across the region. Nonetheless, precursors emitted by local sources, mainly road traffic, play a more important role during O_3_ peaks, with contributions as high as 25 ppb. The potential impact of local measures is higher under unfavorable meteorological conditions associated with regional accumulation patterns. These findings suggest that this modeling system may be used in the future to simulate the potential outcomes of specific emission abatement measures to prevent high-O_3_ episodes in the Madrid metropolitan area.

## 1 Introduction

Air pollution is one of the main environmental problems and is recognized as a global threat to public health. In 2019, 4.2 million people died prematurely worldwide as a result of poor air quality (WHO, 2021). Even in regions that have taken decisive actions to curb emissions, such as Europe, over 300 000 premature deaths (EU27) are currently attributed to air pollution, most of them related to high levels of PM_2.5_ (particles with an aerodynamic diameter of ≤ 2.5 μm) (238 000) and NO_2_ (nitrogen dioxide) (49 000) (EEA, 2022). In recent years, concentrations of many of the regulated pollutants in Europe have decreased as a result of a general reduction in emissions. From 2009 to 2018, the concentration of PM_10_ (particles with an aerodynamic diameter of ≤ 10 μm), PM_2.5_, and NO_2_ diminished on average by 19 %, 22 %, and 18 %–23 % (depending on the air quality monitoring station type), respectively (EEA, 2020). These measures, however, have not reported comparable reductions in ozone (O_3_) ambient concentration levels.

Tropospheric O_3_ is a secondary pollutant formed from photochemical reactions between many different precursors, mainly nitrogen oxides (NO_*x*_ = NO (nitric oxide) + NO_2_) and non-methane volatile organic compounds (VOCs) (Seinfeld and Pandis, 2016; Jenkin and Clemitshaw, 2000; Monks et al., 2015). According to the last European Union (EU) emission inventory report (EEA, 2022), the most important activity sectors regarding O_3_ precursor emissions are the “Road transport” sector (7 % and 37 % of total VOCs and NO_*X*_ emissions, respectively), the “Commercial, institutional and households” sector (15 % and 14 %, respectively), and the “Solvent and product use” sector, representing 42 % of total VOC emissions. Once emitted from urban and industrial areas, these precursors are subsequently transported by the prevailing wind regime (Xu et al., 2011). The atmospheric lifetime of O_3_ depends on numerous variables. In the boundary layer, the atmospheric lifetime of O_3_ is short, roughly 1 or 2 d, depending on the abundance of precursors (Young et al., 2013). In the free troposphere, its lifetime can be up to 2 weeks, time enough to be transported long distances, from the local to the global scale (Monks et al., 2015; Stevenson et al., 2006). In addition to in situ formation, transport of O_3_ from the stratosphere is relevant to explain the tropospheric ozone levels (IPCC, 2007; Hsu et al., 2005). Furthermore, this gas exchange between layers of the atmosphere is expected to increase in the future globally (Meul et al., 2018; Banerjee et al., 2016) due to dynamic and chemical changes in the atmosphere induced by climate change.

Due to these complex dynamics, tropospheric O_3_ levels have not decreased (Jung et al., 2022; Sicard et al., 2023) in accordance with significant NO_*X*_ and VOC emissions reduction (45 % and 41 %, respectively, during 2009–2018). As a result, 12 % of the urban population in Europe is still exposed to high O_3_ concentrations according to EU regulations, with a toll of 24 000 premature annual deaths (EEA, 2022), especially in the Mediterranean basin (Amann et al., 2008; European Environment Agency et al., 2018; EEA, 2020). The share of urban population that suffers from excessive exposure to O_3_ rises to 95 % (EEA, 2022) when the World Health Organization (WHO) guidelines are considered (WHO, 2021). Of note, tropospheric O_3_ produces both short-term (Bates et al., 1972; Bell et al., 2004; Goodman et al., 2018) and long-term health effects (Jerrett et al., 2009; Seltzer et al., 2018), impacting the population living in large urban agglomerations as well as their surroundings. Moreover, it also may have relevant effects on ecosystems (De Andrés et al., 2012; Mills et al., 2011; Harmens et al., 2011) and climate (Sitch et al., 2007; Stocker et al., 2013; IPCC, 2014).

Globally, the latest studies using satellite data suggest that tropospheric O_3_ average levels increased over the past four decades (Ziemke et al., 2019; Gaudel et al., 2018). Paoletti et al. (2014) evaluated observations from monitoring stations in the United States (US) and Europe from 1990 to 2010 and concluded that the O_3_ annual average increased by 7 % yr^−1^ in rural stations and around 12 %yr^−1^–17 %yr^−1^ (US and EU, respectively) in urban stations. However, O_3_ formation is highly non-linear and trends may change depending on the evaluated time period and region, the metric used, and other local factors such as topography or the proximity to the precursor’s emission sources (Reche et al., 2018; Massagué et al., 2023). According to specific studies for the Iberian Peninsula, the trend of the annual average of O_3_ for rural stations during 2004–2012 was not clear (Querol et al., 2014). In contrast, an increasing trend around 1 %yr^−1^–3 %yr^−1^ was observed in all seasons in urban, traffic, and industrial stations. Borge et al. (2019) reported an average increase of 10 μgm^−3^ in daily 8 h maximum O_3_ moving average concentrations (MDA8) for 1993–2017. However, they detected that the highest increase was related to fall and winter (up to 19 μgm^−3^), in agreement with general increases in the oxidation capacity in the atmosphere of the largest urban areas in Europe modeled by Jung et al. (2022).

Nonetheless, the O_3_-forming photochemical activity is largely regulated by weather conditions, especially temperature and solar radiation. For this reason, tropospheric O_3_ formation has a marked seasonal character, with the highest O_3_ values typically recorded in spring and summer (Logan, 1985; Granados-Muñoz and Leblanc, 2016), especially in those locations that are highly influenced by nearby urban areas (Brodin et al., 2010; Carnero et al., 2009) where large amounts of precursors are emitted. Therefore, understanding summertime O_3_ dynamics is more relevant from an air quality management perspective. Furthermore, information on the relative importance of emission sources on ambient levels should be considered when designing plans and measures, especially when they target highly non-linear secondary pollutants such as O_3_ (Cohan and Napelenok, 2011).

There are different source apportionment techniques that may support air pollution research and decision making (Thunis et al., 2019). Approaches based on sensitivities, such as single-perturbation or brute force methods (Borge et al., 2014; Tagaris et al., 2014; Zhang et al., 2022; Qu et al., 2023), may be useful in anticipating the potential effect of a given intervention. However, tagging methods (Grewe et al., 2017; Butler et al., 2018) provide fully mass conservative apportionment at receptors of interest and may be better suited for diagnostic purposes (Borge et al., 2022). These pollution tracking capabilities have been integrated into modern air quality models to provide attribution information together with the standard concentration and deposition output fields, and can be successfully applied to study pollution dynamics (Simon et al., 2018; Pay et al., 2019; Li et al., 2022). This approach may be particularly interesting to describe how O_3_ levels are linked to emission sources under unfavorable meteorological conditions (Cao et al., 2022; Zohdirad et al., 2022) or specific local atmospheric circulation patterns (Zhang et al., 2023) that may lead to high concentration events (Lupaşcu et al., 2022).

This research focuses on the center of the Iberian Peninsula, encompassing the city of Madrid and its surroundings. Consistently with general emission trends in Europe, the emission of the main O_3_ precursors in the Madrid region decreased by 47 % for VOCs and 44 % for NO_*X*_ from 1990 to 2018 (CM, 2021). While recent control measures succeeded in reducing NO_2_ levels (AM, 2022), such emission reductions have, at the same time, substantially impacted urban atmospheric chemistry by modifying its oxidative capacity. Recent studies (Saiz-Lopez et al., 2017; Querol et al., 2016) suggest that O_3_ levels increased in Madrid by 30 %–40 % during 2007–2014. A greater decrease in NO emissions than in NO_2_ emissions (with a subsequent reduction in the NO*/*NO_2_ ratio) may be one of the factors responsible for this response (Querol et al., 2016, 2017; Zaveri et al., 2003; Jhun et al., 2015). The exceedances of the target value for the protection of human health in the region mainly occur in summer, especially under adverse meteorological conditions that have been extensively characterized in previous studies (Querol et al., 2016, 2017, 2018; Millán et al., 2000; Plaza et al., 1997; Pay et al., 2019; Escudero et al., 2019). Preventing these exceedances in the region requires an understanding of the source attribution of O_3_, especially under specific weather patterns that may lead to high pollution levels (Zhang et al., 2023).

In this research, we apply a state-of-the-science air quality model to provide insights into the emission sources and transport patterns which are involved in the formation of tropospheric O_3_ during typical summertime conditions in the Madrid region. In addition to contributing to the scientific understanding of photochemical pollution, the final purpose of this work is to inform the decision-making process needed to design further emission reduction measures in the study area.

## 2 Methodology

### 2.1 Modeling system

The research is supported by a mesoscale modeling system with three main components. Meteorological fields are generated by Weather Research and Forecasting (WRF v3.7.1) (Skamarock and Klemp, 2008). Physics options and parameterizations (Table S1 in the Supplement) are based on previous studies (Borge et al., 2008a; de la Paz et al., 2016), and WRF outputs are postprocessed with the Meteorology–Chemistry Interface Processor (MCIP v5.1) (Otte and Pleim, 2010). Emission processing relies on the US Environmental Protection Agency’s (EPA) Sparse Matrix Operator Kernel System (SMOKE v3.6.5) model (UNC, 2015; Baek and Seppanen, 2018) which has been specifically adapted for the Iberian Peninsula (Borge et al., 2008b, 2014). Biogenic emissions are generated by Model Emissions Gases and Aerosols from Nature (MEGAN v2.1) (Guenther, 2006; Guenther et al., 2012). The third component is the Community Multiscale Air Quality (CMAQ v5.3.2) modeling system (Byun and Schere, 2006; Ching and Byun, 1999). This 3D chemical-transport model (CTM) simultaneously predicts the concentration of all relevant substances considering transport (advection and diffusion), chemical transformation, and deposition. Gas-phase atmospheric chemistry is represented by the Carbon Bond 6 (CB06) (Yarwood et al., 2010) chemical mechanism with chlorine chemistry (CB06r3) (Sarwar et al., 2012; Whitten et al., 2010, Emery et al., 2015) according to SPECIATE 4.0 (Hsu et al., 2006), while the module AERO6 (Appel et al., 2013) is used to describe aerosol dynamics and chemistry. Considering the influence of different scales, from the continental to the regional–urban, on O_3_ levels (Valverde et al., 2016; Pay et al., 2019; Baker et al., 2016; Han et al., 2018), boundary conditions (BCs) are of particular interest. Previous studies in the Iberian Peninsula have demonstrated that O_3_ is particularly sensitive to boundary conditions (Borge et al., 2010). For a more realistic representation of the boundary influence, the mother domain receives 1 h-resolution, dynamic chemical boundary conditions from hemispheric CMAQ (Mathur et al., 2017) simulations.

In this study, the Integrated Source Apportionment Method (ISAM) (Kwok et al., 2013) implemented in CMAQ v5.3.2 (Napelenok, 2020; Shu et al., 2023) is used. ISAM provides apportionment capability of the full concentration and deposition output arrays including the gaseous photochemically active species, such as O_3_, as well as inorganic and organic particulate matter. The CMAQ-ISAM implementation used in this study attributes source identity to secondary pollutants based strictly on reaction stoichiometry with all reactions playing a role that is relevant to the formation and destruction of any species in the chemical mechanism. ISAM is highly customizable for any number of user-specified combinations of emission source sectors and geographical source areas. For O_3_, this implementation differs from the previous ISAM versions (including CMAQ v5.0.2) that attribute the formation of secondary pollutants to source sectors based on chemical regime – NO_*X*_- or VOC-limited O_3_ formation (Kwok et al., 2015) – and from other studies where precursor attribution is directed by the user to either NO_*X*_ or VOC emissions, such as in Butler et al. (2020). Regime-based methods are useful in attributing secondary species that depend on multiple precursors. However, regime determination relies on predefined thresholds of different metrics, often the H_2_O_2_*/*HNO_3_ ratio (Sillman, 1995), that dynamically depend on location and time-specific parameters (Li et al., 2022). By strictly following the stoichiometry of all chemical reactions in the mechanism, this version of ISAM avoids the general necessity of making decisions and assumptions regarding ozone formation regimes. Decisions on tagging method selections are highly dependent on the specific application and the scientific and/or regulatory aims of each individual study. As the needs of the scientific and regulatory communities evolve, so do the apportionment methodologies. Since the conclusion of this work, CMAQ-ISAM has been expanded to include regime-based, stoichiometry-based, and other configuration options. (More information on ISAM as well as sample application and comparison results can be found in Shu et al., 2023.)

### 2.2 Modeling domains

**Figure 1.**
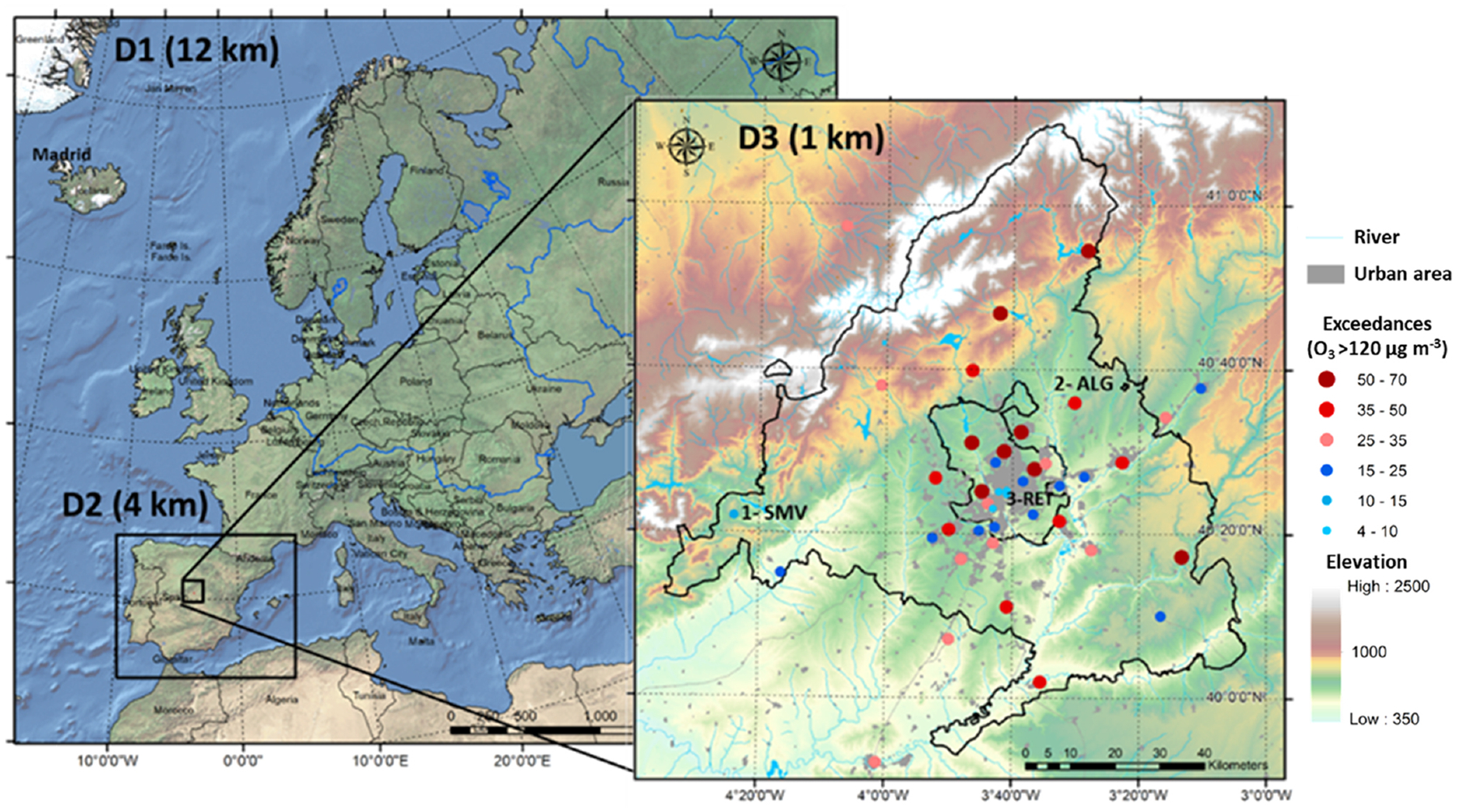
Modeling domains including the location of the air quality monitoring stations within the innermost domain and number of exceedances of the O_3_ target value for protection of human health (MDA8 > 120 μg m^−3^) in 2016.

The three nested domains shown in Fig. 1 were used to perform numerical simulations in this study. This layout is intended to capture medium (Millán et al., 2000) and long-range influences of O_3_ transport (Zhang et al., 2020; Qu et al., 2021; Brook et al., 2013), as well as to provide enough resolution over the area of interest to depict local dynamics (Plaza et al., 1997; Borge et al., 2022). The mother domain (D1) includes Europe and northern Africa with a 12 × 12km spatial resolution, while D2 is centered over the Iberian Peninsula and has a 4 × 4km spatial resolution (Table S2). The innermost domain (D3) used in this study covers Madrid and surrounding areas with 1 km^2^ spatial resolution (136 km in the east–west direction and 144 km in the north–south direction). All three domains have a common 35-level vertical structure covering the whole troposphere with 18 layers within the first kilometer to accurately represent atmospheric processes within the planetary boundary layer (Borge et al., 2010).

The region has a continental Mediterranean climate with an annual mean temperature of 14.6 °C and 367 mm of accumulated precipitation with a typical summer drought (https://www.madrid.org/iestadis/fijas/coyuntu/otros/cltempe.htm; last access: 10 May 2023). The Central Range (Sierra de Guadarrama), with maximum elevations of 2500 m above sea level (m a.s.l.), crosses the D3 modeling domain in the NE–SW direction and divides it into two main regions: the northern and southern plateaus of the Iberian Peninsula. The southern half of the domain, where the city of Madrid (with an average elevation of 657 m) is located, features the Tajo River basin. This topography configures a dominant wind circulation along the NE–SW direction and enhances anticyclonic stagnation conditions (Plaza et al., 1997; Querol et al., 2018) usually induced by the semi-permanent Azores High (García et al., 2002). O_3_ formation typically peaks with high temperature and solar radiation under stagnation conditions (Querol et al., 2018; Reche et al., 2018; Garrido-Pérez et al., 2020) that often occur in summer.

### 2.3 Temporal domain

Model simulations were completed for July 2016 using a previous 3 d period as model spin-up. According to the Spanish Meteorological Agency (AEMET, 2017) it was an unusually warm month (with an average temperature of 25.5 °C) and the fourth hottest month of July since 1961 in the Iberian Peninsula. It was also a dry month, with 13 % less precipitation than the average of the month in the 1981–2010 reference period. Considering the meteorological trends in this region (Borge et al., 2019), it can be regarded as a representative summer period for modern weather conditions. More importantly, this period was selected because of an intensive experimental campaign carried out to characterize ozone episodes in Madrid and surroundings (Reche et al., 2018). This period was thoroughly analyzed by Querol et al. (2018), who identified two typical circulation patterns associated with venting and accumulation episodes. The latter are characterized by weak wind forcing (wind speed <4–5 ms^−1^), stable conditions, and air stagnation that favors O_3_ local formation. Oppositely, stronger winds (> 7 ms^−1^) promote advection and prevent the reaching of O_3_ peaks under venting conditions.

During this period (2016), 26 out of the 42 air quality monitoring stations in the innermost (D3) modeling domain (Fig. 1) recorded exceedances of the concentration threshold related to the O_3_ target value for the protection of human health (MDA8 > 120 μg m^−3^). The highest number of exceedances (up to 359 in the month, 47 % of total annual exceedances) were found around the Madrid metropolitan area, in the city outskirts. Of note, no exceedances of the MDA8 were recorded for downtown Madrid.

### 2.4 Emission sources for the apportionment analysis

Emissions for this modeling exercise result from the combination of the official national (MMA, 2018), regional (CM, 2021), and Madrid’s city local inventory (AM, 2021). These inventories are compiled according to the EMEP/EEA standardized methodology (EEA, 2019) and are conveniently adapted, spatiotemporally resolved for modeling purposes (Borge et al., 2008b, 2018) and consistently combined for the different modeling domains (Borge et al., 2014).

Emissions from power generation and industrial activities (SNAP01, SNAP03, and SNAP04 groups according to the Selected Nomenclature for Air Pollution nomenclature) were merged due to their limited presence in this modeling domain. Since emissions from agriculture (SNAP10) in the region are mainly significant for VOCs from plants, they have been tagged alongside biogenic VOC (BVOC) emissions from vegetation (SNAP11) (labeled as “S10-S11” for SNAP10–11 in Fig. 12). Soil-NO_*X*_ emissions provided by MEGAN 2.1 (Yienger and Levy, 1995) are also included in this group, and their share to total NO_*X*_ emissions is around 4 % in this period (July 2016), consistent with MEGAN results reported on the European scale (Visser et al., 2019).

**Figure 2.**
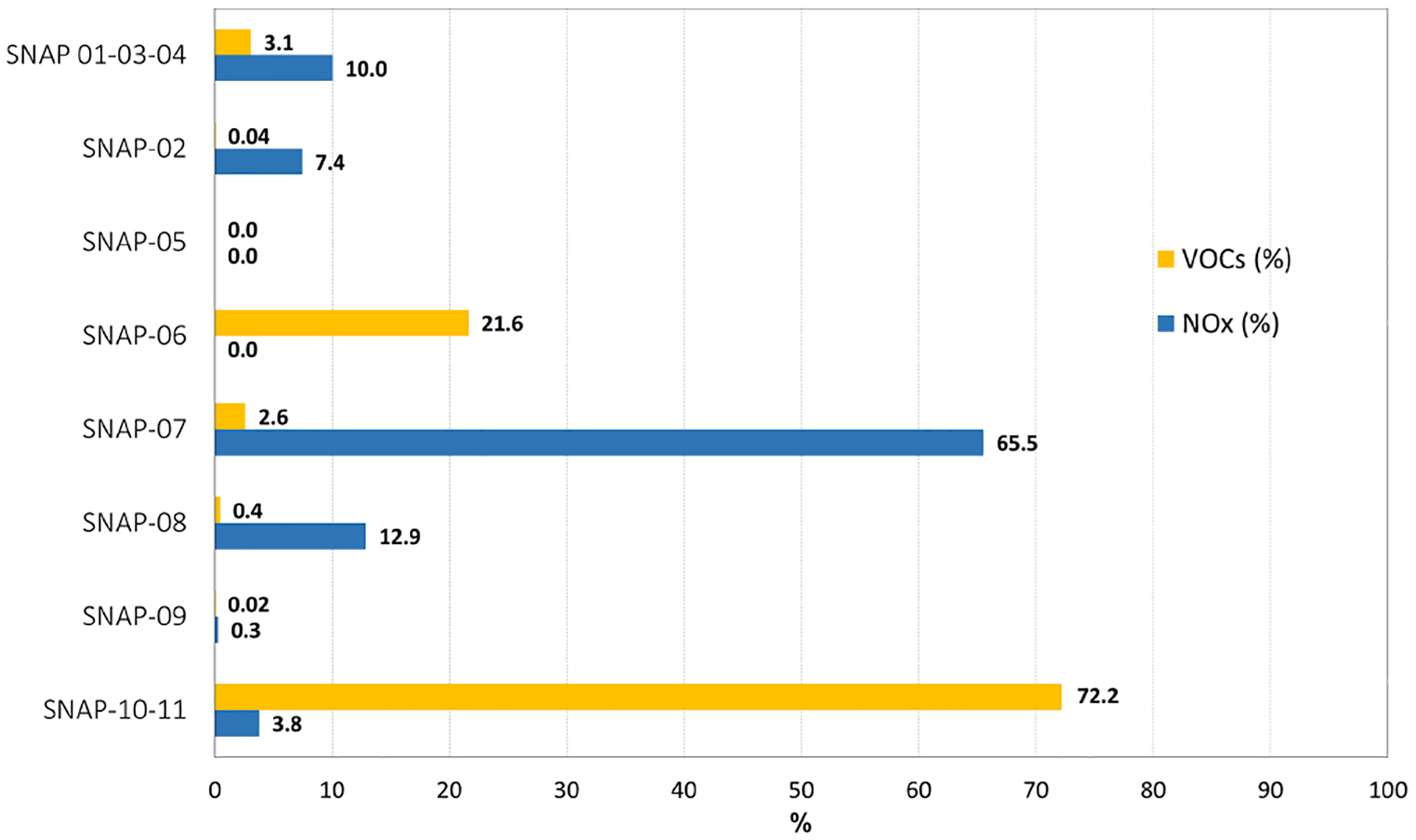
NO_*X*_ and VOC emissions of tagged sectors for July 2016 (percentage over total emissions in the modeling domain) for the source apportionment analysis.

Consequently, eight emission sources (Table 1) were tagged for the source apportionment analysis of ambient O_3_ in the region. The share of NO_*X*_ and VOC emissions of each of them for July 2016 is summarized in Fig. 2. The emission breakdown on an annual basis can be found elsewhere (Borge et al., 2022). Figure 2 shows that they account for the totality of emissions in the modeling domain and identifies road traffic (SNAP07) as the main source of NO_*X*_ (66 % of total emissions), followed by other mobile sources (SNAP08). Since emissions from the residential, commercial, and institutional sector (SNAP02) occur almost exclusively in winter, the contribution from this sector is relatively small (around 7 %) and is related to combustion units in agriculture and forestry. VOC emissions are dominated in this period by emissions from plants. The combined contribution of forests and agriculture represents 72 % of total VOCs. Solvent use (SNAP06) is the main anthropogenic source of this O_3_ precursor with a total share of 22 % (nearly 80 % of anthropogenic VOC emissions).

In addition to the attribution of O_3_ ambient levels to the emissions within the modeling domain, hereinafter referred to as local sources, the contribution of boundary conditions (BCs) and initial conditions (ICs) are estimated in this study. Considering the typical O_3_ daily patterns and the variability of circulation patterns, the latter refers to the initial mixing ratios on a daily (24 h) basis, i.e., each day is run separately using the outputs from the previous day as ICs. This is different from most previous source apportionment studies that analyze shorter periods (Pay et al., 2019) or specific high concentration events (Lupaşcu et al., 2022; Zhang et al., 2022). While this may hinder the comparability of our results, this methodological option may be appropriate considering the temporal span of the period analyzed (a whole month), the typical diurnal cycle of O_3_, and the goal of characterizing this attribution under specific meteorological conditions. This helps in understanding differences on O_3_ source apportionment depending on regional circulation patterns (Zhang et al., 2023) and explicitly considering the influence of vertical transport of O_3_ from residual layers from previous days that may lead to rapid increases in O_3_ concentrations near the surface (Qu et al., 2023, and references therein). Therefore, this approach may be better suited to provide useful information for decision making, especially for the design of short-term action plans intended to control ozone peaks.

## 3 Results

The results are presented in four subsections. First, the main features of the simulated period and model performance are presented. Then, an overview of the source apportionment analysis carried out in the study area for the whole month is discussed. Next, this same analysis is performed for two specific days representative of different circulation patterns defined by Querol et al. (2018): advective pattern (13 July) and accumulation pattern (27 July). Additional information for 20 July and 6 July, identified by Querol et al. (2018) as advective and accumulation days, respectively, is provided in the Supplement. Finally, the temporal patterns of the O_3_ apportionment are examined at the location of the air quality monitoring stations within the simulation domain. Aggregated results by station type are discussed in Sect. 3.4, while the results for different geographical areas relative to the location of Madrid city (quadrants) are presented in the Supplement.

### 3.1 Ozone levels during the study period and model evaluation

**Figure 3.**
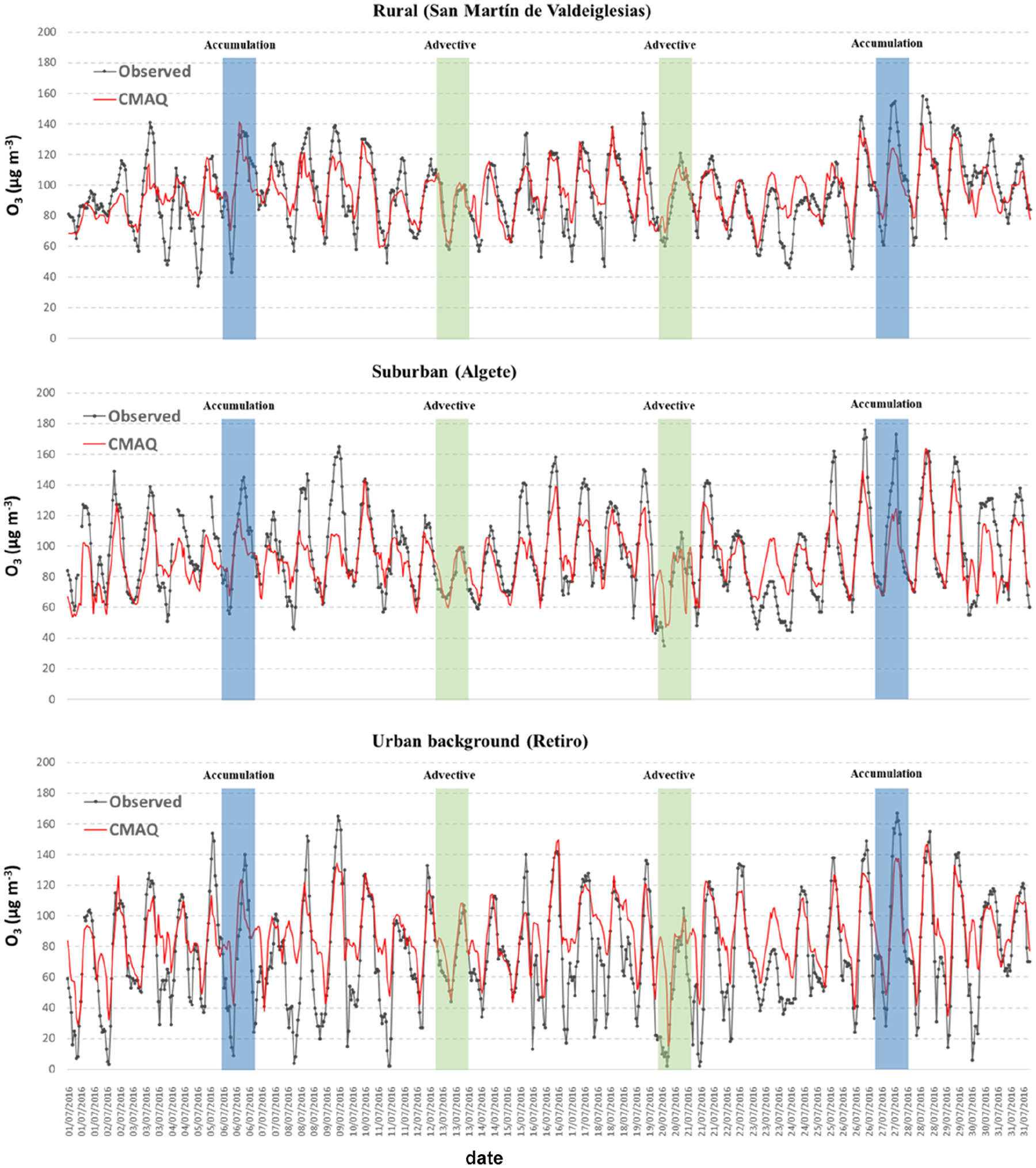
Observed and predicted concentration series for selected locations (1 – SMV: a rural location in the southwestern area of Madrid; 2 – ALG: a suburban location in the northeastern area of Madrid; and 3 – RET: an urban background site in Madrid’s city center).

While this period was hotter and dryer than most of recent summers, July 2016 may be representative of typical summer conditions in the Madrid region and included a concatenation of characteristic local circulation patterns (Plaza et al., 1997) with direct implications on ground-level O_3_ (Querol et al., 2018; Escudero et al., 2019). Figure 3 presents both observed and modeled concentration series at representative points (Fig. 1), and shows the venting and accumulation days identified in Querol et al. (2018). The time series demonstrate that O_3_ levels are significantly lower under venting conditions, although significant differences are found depending on the location, which supports the need to use high-resolution modeling systems to analyze pollution dynamics in the Madrid region. On the other hand, accumulation patterns tend to produce higher concentrations (up to 175 μg m^−3^), especially during 27 July.

It can be observed that the model is able to reproduce the temporal patterns, as confirmed by the high correlation coefficients (*r*) and index of agreement (IOA) shown in Table 2. The statistical evaluation demonstrates a reasonable model performance yielding better statistical results than recent simulation studies in this domain. Pay et al. (2019) reported an aggregated correlation coefficient of 0.66 and mean bias (MB) of 22.5 ug m^−3^ for the central region of the Iberian Peninsula. In this study, we obtained an average *r* value of 0.74 and an MB of 6.2 ug m^−3^. Of note, 95.2 % and 66.7 % of the *r* values for the locations of the 42 monitoring stations used in this study are larger than 0.6 and 0.7, respectively while the overall normalized mean bias (NMB) is only 9 %. The results for a series of common statistics (Borge et al., 2010) for each of the monitoring sites in our modeling domain can be found in Table S3. The model, however, may have some difficulties capturing the amplitude of observed O_3_ series and fails to accurately reproduce concentration peaks on some days. This is evidenced by the relatively large error in comparison with the bias (23 % and 9 %, respectively, on average over the 42 monitoring stations in the modeling domain). In Table S4, we present a separate model performance assessment for accumulation and advective patterns showing that the main differences among them relate to errors, both mean gross error (MGE) and root mean squared error (RMSE), that are systematically higher for accumulation periods. This may be related to the limitations of the meteorological model in depicting atmospheric circulation during stagnation conditions suggested by Pay et al. (2019). Even when WRF was found to outperform other models for this particular episode (Escudero et al., 2019), the ability to reproduce wind direction and wind speed clearly deteriorates for accumulation periods, as shown in Table S5.

As expected, results are poorer for urban background and urban traffic locations, since the typical spatiotemporal representativeness of the measurements in such locations is not comparable with that of a mesoscale modeling system, even with 1 km^2^ spatial resolution.

### 3.2 Spatial analysis of the source apportionment assessment

In this section, we discuss the contribution to ground-level O_3_ of the tagged sources (Table 1) both for monthly average and high values (illustrated by the 90th percentile (P90)). O_3_ apportionment focuses on anthropogenic sources since they have more interest from the point of view of possible abatement measures (Oliveira et al., 2023) and have a larger contribution than that of SNAP10–11 (below 4 % of total O_3_ levels in this period). However, it is not a negligible apportionment since these groups account for 27 % (monthly mean) and 22 % (P90) of total O_3_ averaged over the Madrid region when BCs and ICs are not considered (Fig. S1 in the Supplement). Non-anthropogenic emissions have been reported to play an important role in atmospheric photochemistry and they interact with man-made emissions, so they need to be considered in the process of designing policies to reduce tropospheric O_3_ levels. Therefore, we discuss the potential role of emissions from agriculture and nature as well.

#### 3.2.1 Non-anthropogenic sources

According to our results, the combined contribution of SNAP10–11 represents around 21 % and 28 % of that of local anthropogenic emissions to the monthly mean and P90. This is a similar share to that reported by Sartelet et al. (2012) at the European scale. As well as Collet et al. (2018), they argue that the influence of BVOC becomes stronger on VOC-limited areas which is consistent with our findings (Fig. S2) since the Madrid region is predominantly NO_*X*_ limited in summer, except for the metropolitan area of Madrid city and surroundings, which remain VOC limited all year-round (Jung et al., 2022, 2023). Pay et al. (2019) did not quantify explicitly the contribution of biogenic emissions to ozone in the Iberian Peninsula. However, the contribution of “other”, which included emissions from SNAP11 along with other sectors, was around 5 % in the center of the Iberian Peninsula, even though biogenic emissions represent a large fraction of total VOCs.

The contribution of BVOC to ozone levels in Europe reported by Tagaris et al. (2014), Karamchandani et al. (2017), and Zohdirad et al. (2022) are slightly larger (below 6 %) and are even more according to some source apportionment at the global scale for this latitude (Grewe et al., 2017; Butler et al., 2020). It should be noted that different experimental design and apportionment algorithms would lead to significant differences (Zhang et al., 2017; Borge et al., 2022) preventing the direct comparison of the results from different studies. In addition to the apportionment methodology itself, the results may differ depending on the emission inventory used, the modeling scale and resolution, temporal span, and sources tagging scheme. Nonetheless, the contribution of biogenic emissions found in our work is not remarkably different from that previously reported, especially for this same geographical area.

Previous studies suggested that relatively low contributions of biogenic VOCs to O_3_ levels may relate to underestimations of isoprene levels (Lupaşcu et al., 2022), a very relevant species for O_3_ chemistry (Dunker et al., 2016) that constitutes more than 25 % of global biogenic VOC emissions Guenther et al. (2012). Nonetheless, it is widely recognized that BVOC emission estimates involve large uncertainties (Poupkou et al., 2010; Wang et al., 2017; Zhang et al., 2017), and the MEGAN model used in this study has been found to overestimate isoprene emissions (Wang et al., 2017, and references therein). According to our inventory, isoprene represents 48 % of total BVOC. While isoprene ambient measurements are not made routinely, Querol et al. (2018) recorded an average level of isoprene around 0.2 ppb in Majadahonda, a suburban site ~ 15 km away (in the W–NW direction) from downtown Madrid, between 5 July and 19 July 2016. That is in relatively good agreement with the results of CMAQ in our simulation, which predicted slightly less than 0.1 ppb for that location and period, as well as reproduced quite accurately the average daily pattern (see Fig. S3).

Arguably, the relatively low contribution of BVOC in our study and previous studies in this area (Valverde et al., 2016; Pay et al., 2019) may be a consequence of the underestimation of isoprene mixing ratios. However, that is compatible with the stronger influence of other anthropogenic VOC species reported elsewhere. Querol et al. (2018) estimated the total ozone formation potential (OFP) applying the maximum incremental reactivity (MIR) proposed by Carter (2009) to the VOC measurements made in their campaign for the same period and location as those in our study. Based on this methodology, they identified formaldehyde as the single most important compound (35.5 % of total OFP), while isoprene was ranked seventh with an OFP below 5 %. By family, primary BVOCs represented 6 % of total OFP on average during the experimental campaigns in this period. Similar studies elsewhere (e.g., Meng et al., 2022, in the Pearl River Delta region) have concluded as well that the ozone formation potential of BVOCs is lower than that of anthropogenic VOCs applying a similar reactivity scale (Carter and Atkinson, 1989). That may be consistent with the apparent insensitivity of O_3_ to isoprene emissions reported in other studies (Simpson, 1995; Jiang et al., 2019; Ciccioli et al., 2023).

Of note, SNAP10–11 include NO_*X*_ emissions from soils (see Sect. 2.4). Although they represent less than 4 % of total NO_*X*_ emissions in the domain, they may be underestimated by MEGAN (Visser et al., 2019). According to other studies, for example, Weng et al. (2020), emissions from agricultural soils may be substantially higher and could pose a significant constraint in the control of O_3_ levels (Lu et al., 2021). Methods to reduce the uncertainty in NO_*X*_ emissions estimates from soils as well as their role in O_3_ control policies specifically for this region may be addressed in future research.

Other non-controllable sources include stratospheric ozone, also tagged in this study (ST in Fig. 12). This source informs about the influence of vertical injections on ground-level O_3_ levels (Hsu et al., 2005) and the potential contribution reported in this region for specific extraordinary ozone levels (San José et al., 2005). Pay et al. (2019) hypothesize that stratosphere–troposphere exchange (STE) may have played a significant role towards the end of July 2016 in the Iberian Peninsula. According to our results, however, the direct transport of O_3_ from the stratosphere in our modeling domain was negligible in this period, with 1 h maximum contributions below 0.4 ppb in the southwest end of the Madrid region (see Fig. S4). This contrasts with remarkably higher contributions reported in other areas of Europe (Lupaşcu et al., 2022) and those from global simulations for similar latitudes (Butler et al., 2018). It should be noted that here we account for O_3_ STE exclusively within our innermost nested domain and part of the O_3_ attributed to BCs may be related to contributions from the stratosphere in other regions.

#### 3.2.2 Anthropogenic sources

**Figure 4.**
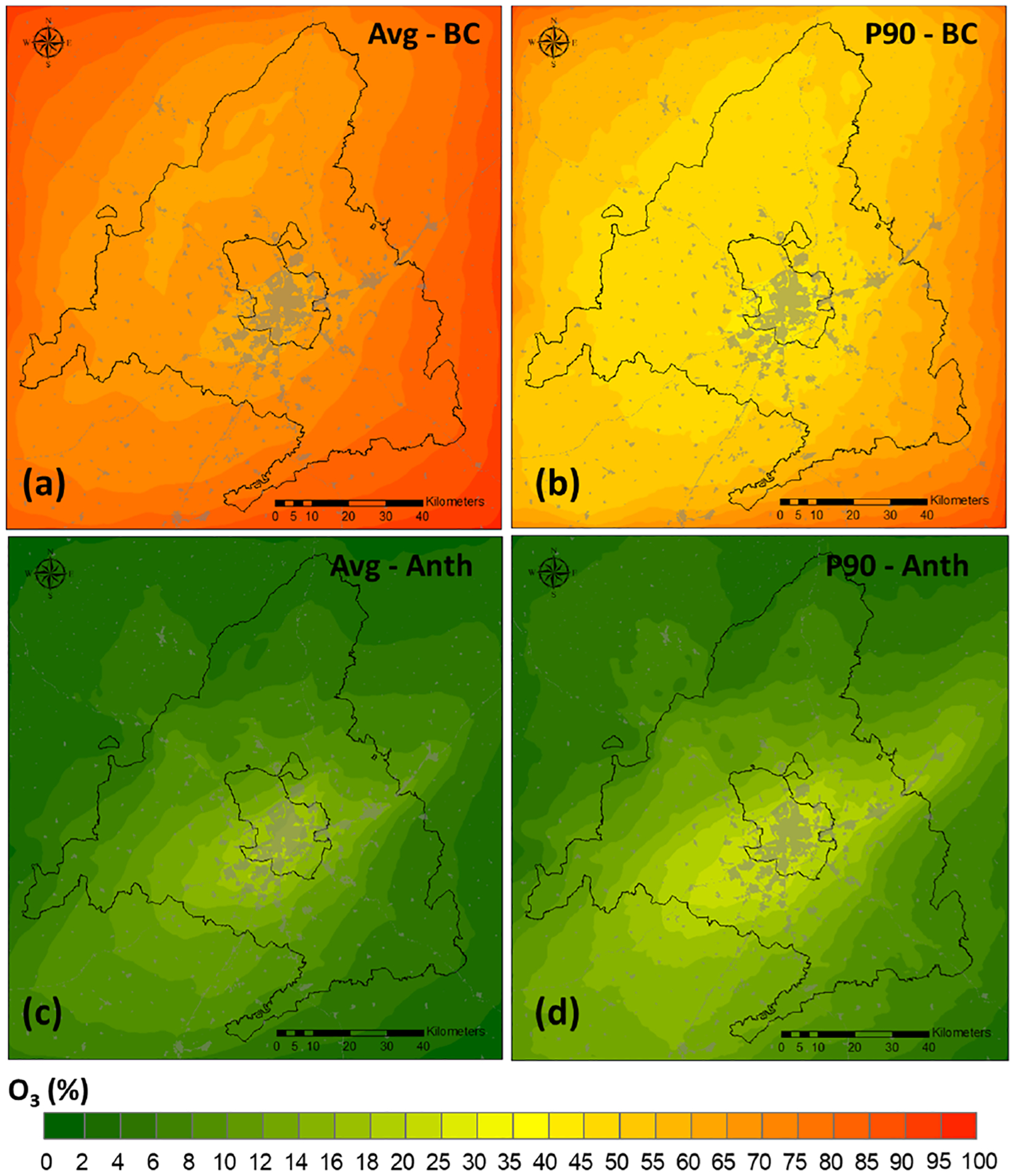
Contribution (%) of BCs to O_3_ concentration: (**a**) monthly average and (**b**) 90th percentile. Contribution (%) of local anthropogenic emissions to (**c**) monthly average and (**d**) 90th percentile.

Figure 4 shows the contribution to ground-level O_3_ of the BCs and that of all local anthropogenic emissions combined for both monthly average and high values (illustrated by the 90th percentile (P90)). In both cases BCs are the largest contributor. This is consistent with previous studies that have identified boundary conditions as the dominant contribution to ground-level O_3_, e.g., Pay et al. (2019) for the Iberian Peninsula, Collet et al. (2018) for the USA, or de la Paz et al. (2020) for Madrid specifically. However, the weight of each of the sources on both metrics is different. On average, 70 % of the mean O_3_ levels in the Madrid region comes from BCs (Fig. 4a), while for P90, the contribution from BCs is considerably smaller, around 50 % (Fig. 4b).

The maximum anthropogenic contribution for the monthly average (Fig. 4c) reaches 17 % (7.5 ppb in absolute terms), with a mean contribution of 8.7 % over the whole Madrid region (Fig. S1). Regarding P90 (Fig. 4d), the maximum contribution of local anthropogenic emissions is 28 % (in the center and southwest of the Madrid municipality), around 22 ppb in absolute terms. This corresponds to a spatially averaged contribution of 12.2 % over the Madrid region (Fig. S1), which corresponds to an absolute value around 11 ppb. Despite the general dominance of BCs on O_3_ levels, these results point out that the relevance of local emissions (Fig. 2) is higher for O_3_ peaks, a finding consistent with those of previous studies (Valverde et al., 2016; Qu et al., 2023).

**Figure 5.**
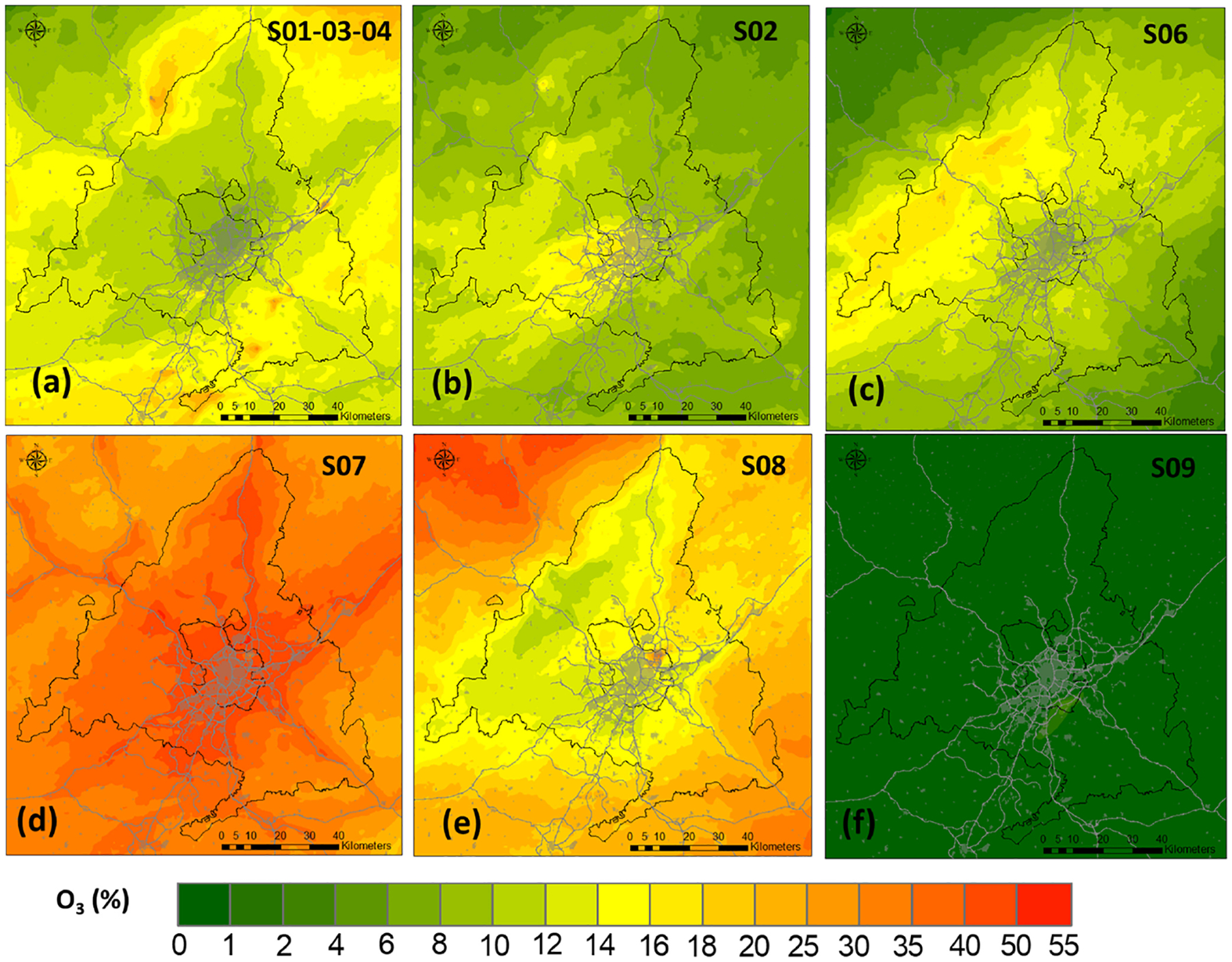
Percentage contribution to the 1 h O_3_ 90th percentile of the main emitting sectors with respect to the total anthropogenic contribution.

Figure 5 shows the apportionment to P90 of each emission sector for local sources. Consistent with Valverde et al. (2016) and Pay et al. (2019), our results clearly identify road transport (SNAP 07) as the most influential sector, contributing 41 % to P90 on average over the Madrid region. The contribution of this sector (relative to local sources) reaches values up to 55% in the proximity of the main communication routes (Fig. 5d). In absolute terms, this means an average contribution of 5 ppb and a maximum one of 11 ppb (Fig. S5). The next sector with the highest contribution relates to off-road mobile sources (SNAP08), with an average contribution in the Madrid region of 17 % (1.8 ppb) and a maximum of 8 ppb in the vicinity of the Adolfo Suárez Madrid–Barajas airport. This suggests that NO_*X*_ emissions play a more important role than VOC emissions in the photochemical production of ozone, in concurrence with previous source apportionment studies (Dunker et al., 2016; Butler et al., 2018; de la Paz et al., 2020). Nonetheless, the importance of controlling anthropogenic VOC emissions to prevent high-O_3_ episodes has been noted in previous studies (Cao et al., 2022), even in regions with strong biogenic emissions (Coggon et al., 2021). In addition to the contribution of BVOC previously discussed, anthropogenic VOC had also an influence on O_3_ levels during July 2016 in the Madrid region (see Fig. S2). While the spatially averaged attribution of SNAP06 to P90 is only 1.5 ppb with maximum contributions of 3 ppb at specific locations (southwest of Madrid as shown in Figs. S2 and S5), emissions from the use of solvents and other products can reach values up to 20 % of total anthropogenic contributions to O_3_ P90 (Fig. 5c). This is comparable to the contribution of all industrial sources combined (SNAP01–03-04). This may be related to the high OFP of aromatics within SNAP06 VOC (Meng et al., 2022) and is consistent with the findings of Oliveira et al. (2023) that attributed 64 % of total OFP to the solvent sector (relative to that of total anthropogenic VOC) in densely urbanized areas such as Madrid. Coggon et al. (2021) also found that consumer and industrial products (included in SNAP06 group) are important precursors of ozone in urban areas, which typically present a VOC-sensitive regime. Nonetheless, they found that O_3_ formation may take a few hours and the maximum contributions of VOC emitted in New York City occur a few tens of kilometers away, close to NO_*X*_-limited areas. Our high-resolution analysis indicates that a similar process may take place in Madrid too. The rest of the sectors analyzed (SNAP05 and SNAP09) have negligible contributions (around 0.05 ppb on average over the Madrid region).

If the analysis is done on a daily basis, it is worth noting the significance of the initial conditions (ICs) as well, with a spatially averaged contribution of 19 % and 34 % to the monthly average and P90 O_3_ levels, respectively (Fig. S1). However, the role of ICs is more relevant in analyzing how meteorological conditions may affect source apportionment. Of note, in this study “ICs” refers to O_3_ from the previous 24 h period. Consequently, the effect of ICs on O_3_ does not necessarily diminish throughout the month. Instead, we found that the influence of ICs relates mainly to regional circulation patterns. We elaborate on this in the following sections.

### 3.3 Source apportionment assessment under characteristic circulation patterns

The study of the influence of meteorology on the O_3_ ambient levels is carried out by analyzing the results for specific days representative of the two circulation patterns. Querol et al. (2018) identified an advective pattern for 13 and 20 July and an accumulation pattern for 6 and 27 July. In this section, we examine the source apportionment for those days (days 13 and 27 in more detail) to test the hypothesis that local atmospheric conditions may induce a significant difference in O_3_ attributions, as reported elsewhere (Zhang et al., 2023).

**Figure 6.**
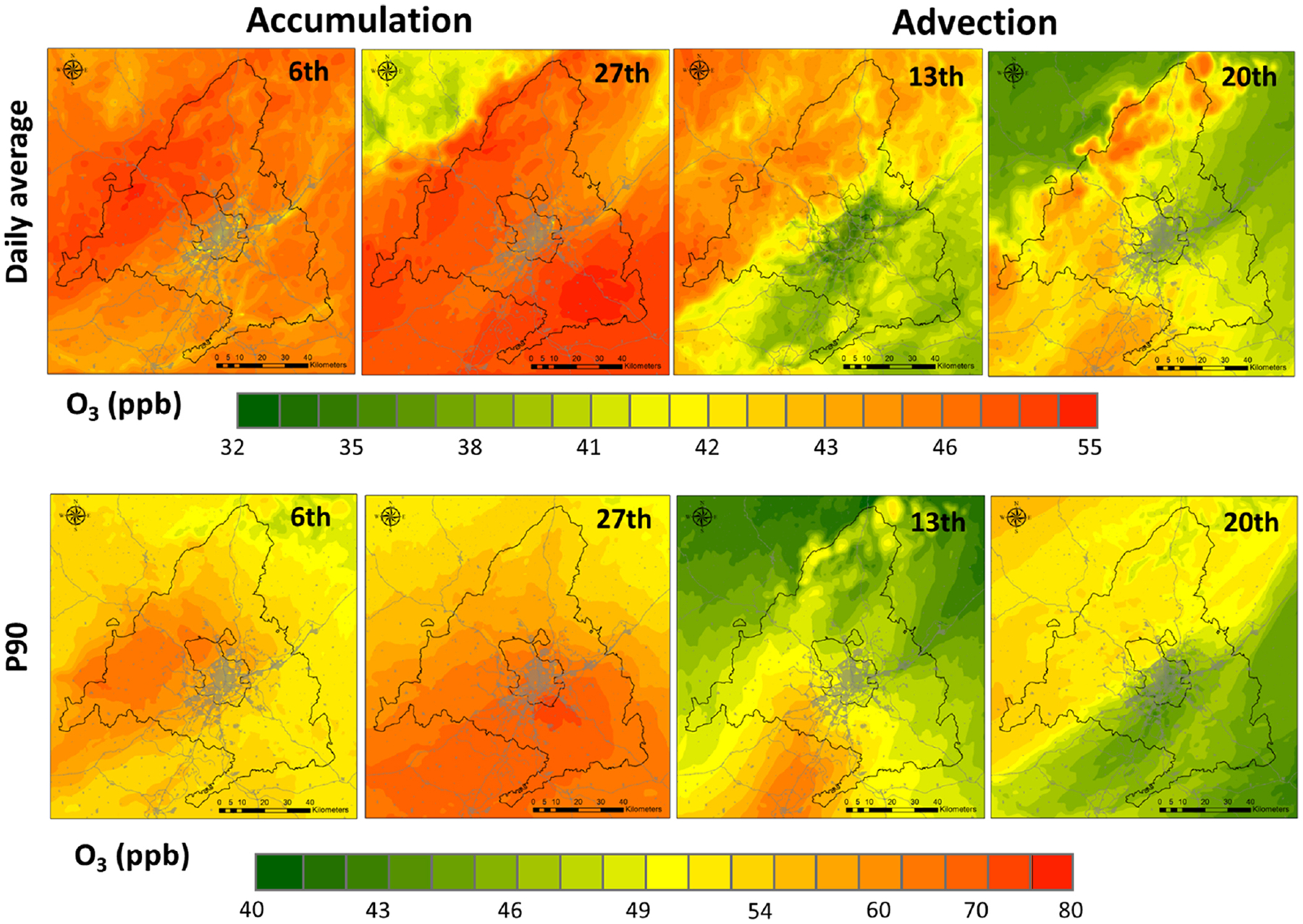
Daily mean (top) and 90th percentile (bottom) of O_3_ levels (ppb) during accumulation (6 and 27 July 2016) and advective (13 and 20 July 2016) periods.

Figure 6 shows the daily average and the P90 of hourly O_3_ levels during the accumulation and advective episodes. It is observed that during accumulation days (days 6 and 27), mixing ratios averaged over the Madrid region were 13 %–20 % higher than those of advective periods (days 13 and 20) and, although not shown, around 4 %–8 % higher than the monthly average. Regarding the maxima, the average P90 (third highest hourly mixing ratio for a given day) during the accumulation periods in the Madrid region may be 25 % higher than that of the ventilation periods.

#### 3.3.1 Accumulation pattern

**Figure 7.**
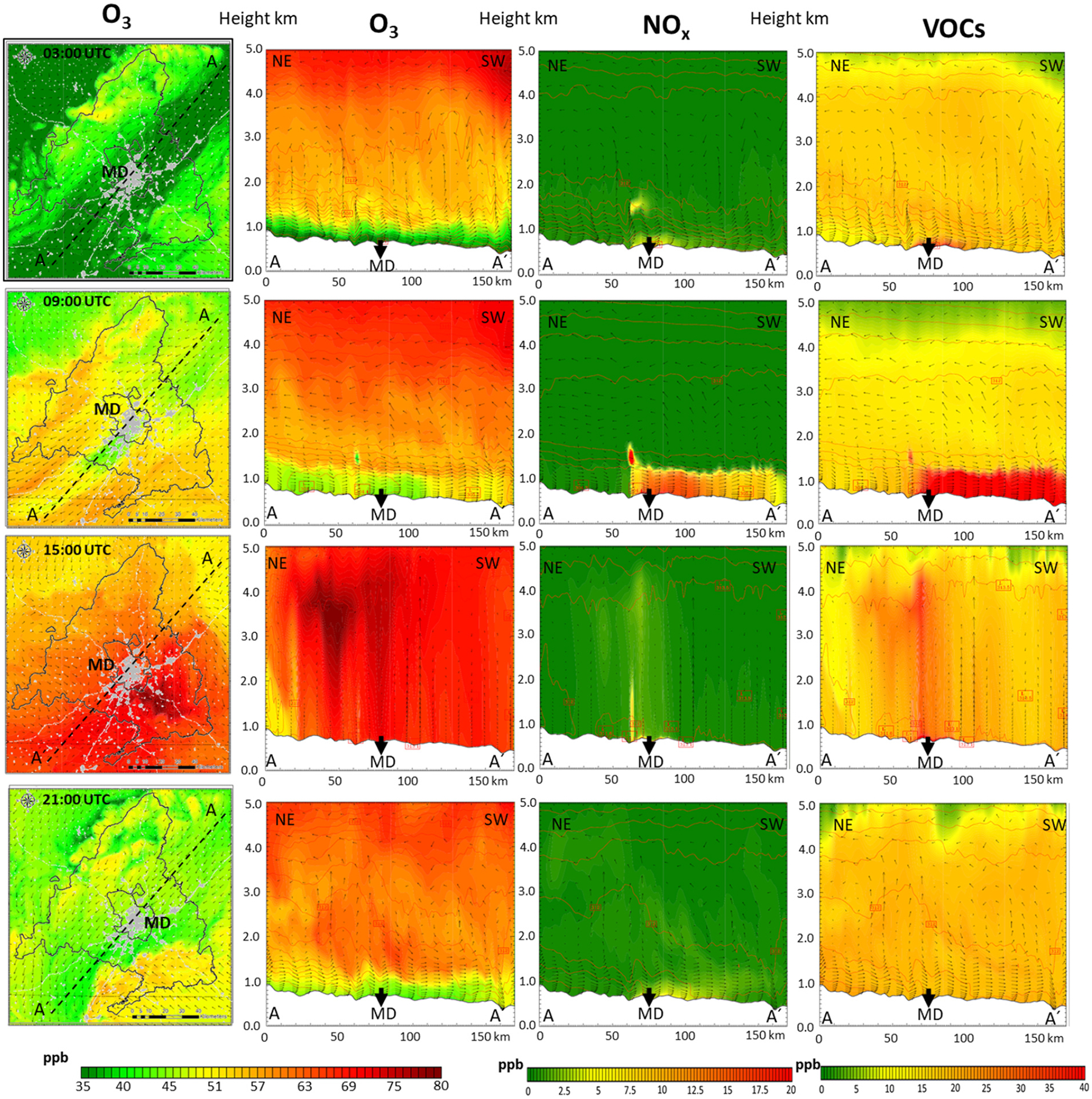
Accumulation period: evolution during 27 July 2016. From left to right: plan view and NE–SW cross section (up to 5 km height) O_3_ mixing ratios (ppb), NO_*X*_ (ppb), and VOCs (ppb) at the 03:00, 09:00; 15:00, 21:00 UTC hours. MD: Madrid city.

Consistent with previous studies that highlight the impact of meteorological conditions on O_3_ (Nguyen et al., 2022), modeling results show that accumulation days are especially relevant regarding the potential impacts on health and vegetation, and a deeper analysis of pollution dynamics under those conditions is of interest. Figure 7 shows the hourly evolution (03:00, 09:00; 15:00, 21:00 UTC) of surface O_3_ mixing ratios during the day 27 (day 6 is shown in Fig. S7), along with O_3_, NO_*X*_, and VOC vertical levels up to 5 km height for a NE–SW cross section, related to the dominant wind directions. (The same results for a perpendicular SE–NW cross section are shown in Fig. S8.)

A low O_3_ mixing ratio surface layer (around 40 ppb) can be clearly seen for the early hours of the day (03:00 UTC, 05:00 LT). This relates to a shallow planetary boundary layer (PBL) (a few hundred meters high) and weak winds (1–2 ms^−1^) from the NE. Around 06:00 UTC (08:00 LT), the main emitting sectors (such as road transport) begin to emit O_3_ precursors (see Quaassdorff et al., 2016) for characteristic emission temporal profiles. The prevailing surface wind directs the urban plume towards the SW and the southern slope of the Sierra de Guadarrama (Fig. S6). Of note, the wind direction aloft is the opposite, in accordance with recirculation processes reported for this domain (Plaza et al., 1997). As the day progresses (09:00 UTC, 11:00 LT), the PBL height grows (up to 1.5 km) as radiation and temperature increase, mixing O_3_ vertically. At the same time, the emissions of precursors (concentrated in Madrid city) lead to an increase in the local production of O_3_ in the plume, more evidently in the rural areas (NO_*X*_-limited regions) in the leeward side of the city. On the contrary, in the vicinity of high NO_*X*_ emission intensity areas, O_3_ is consumed by NO through the reaction NO + O_3_ → NO_2_ + O_2_, a titration effect documented in previous studies (Saiz-Lopez et at., 2017).

**Figure 8.**
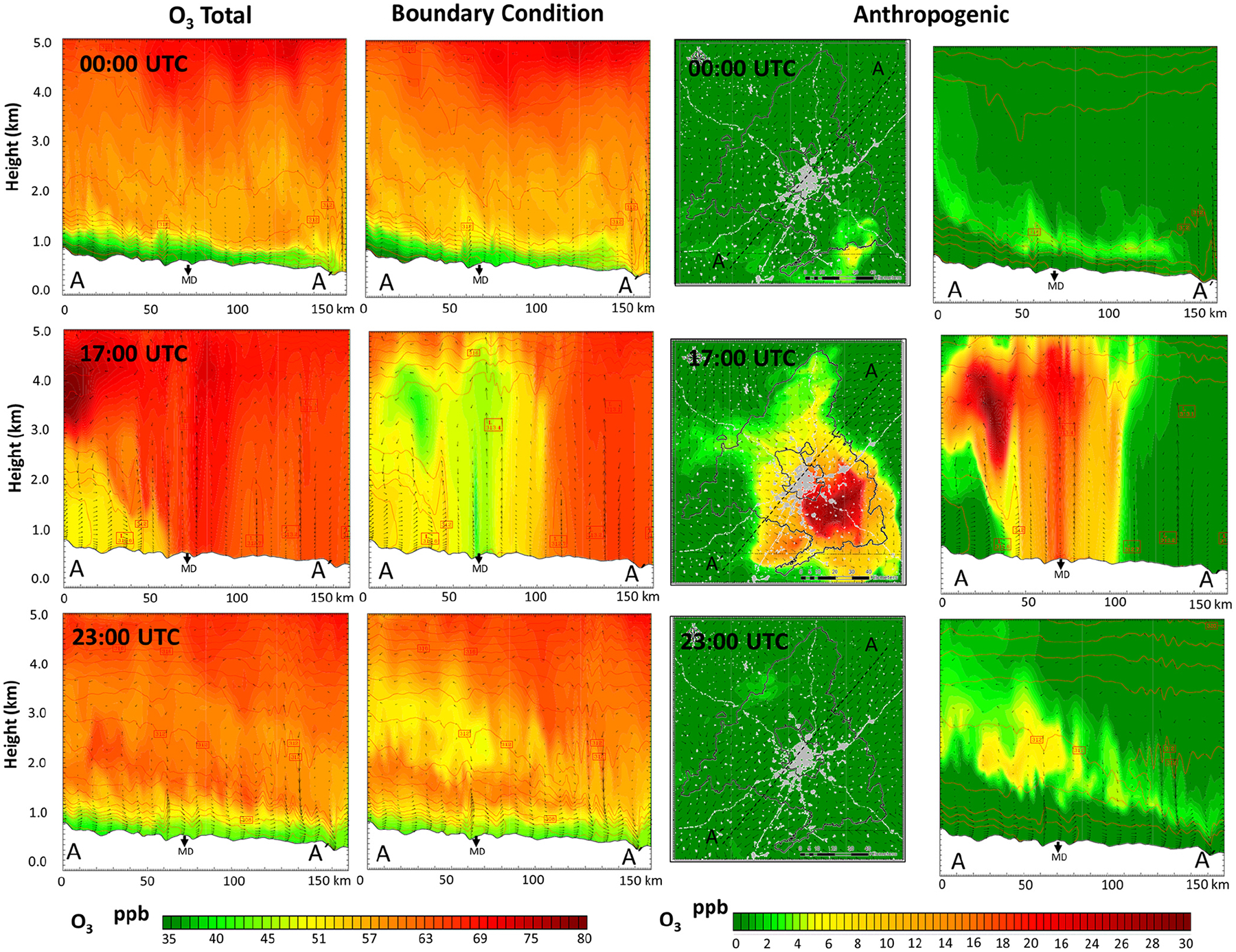
Hourly O_3_ mixing ratio profiles (at 00:00, 17:00; 23:00 UTC) for the NE–SW cross section as well as the contribution of BCs and anthropogenic local emissions on 27 July 2016 (accumulation). MD: Madrid city.

**Figure 9.**
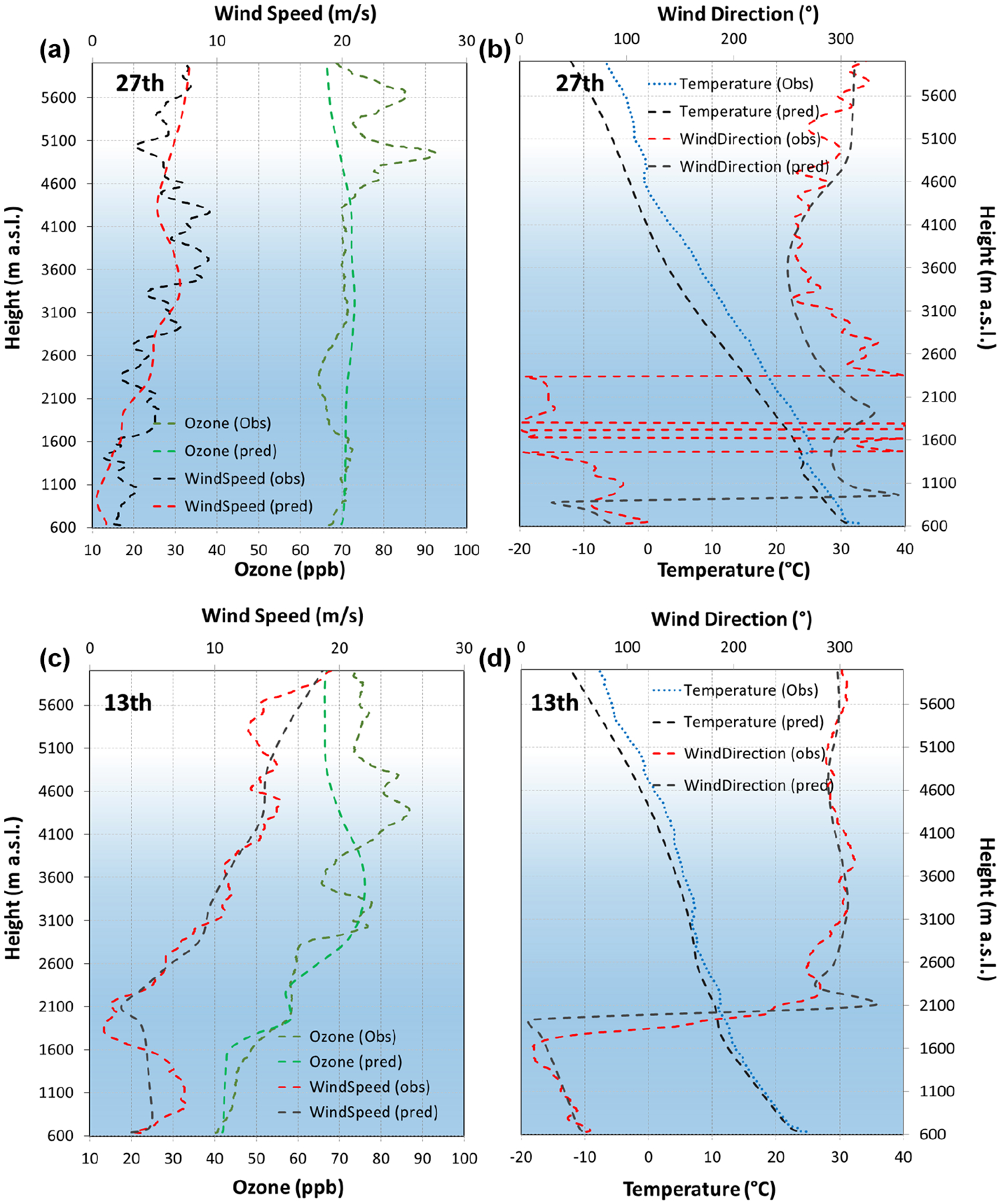
Vertical profiles (12:00 UTC) of O_3_ mixing ratios, temperature, wind speed, and wind direction for 27 July (accumulation, up) and 13 July (advective, down).

Over the following midday hours (09:00–15:00 UTC, 11:00–17:00 LT) the PBL further develops and a vertical homogenization process occurs. There is a deep vertical mixing of newly formed ozone with O_3_-enriched upper layers generated in previous days (Querol et al., 2018; Escudero et al., 2019). As illustrated in Fig. 8, there is a first O_3_ reservoir located around 1500 m altitude (at 00:00 UTC, 02:00 LT) that relates mainly to local sources and contributes with 2–8 ppb, while higher O_3_ reservoirs (around 4000 m a.s.l.) relate to BCs and have a considerably higher contribution (50–75 ppb). Around 15:00 UTC (17:00 LT) the PBL reaches 3000–4000 m in accumulation periods and O_3_ levels up to 75–80 ppb are found (Fig. 7). This dynamic is compatible with the ozone sounding (https://woudc.org/data/explore.php; last access: 10 November 2022) included in Fig. 9, which shows a very constant O_3_ value around 65–70 ppb from the surface to 4000 m a.s.l.

Later, around 17:00 UTC, the local O_3_ production from anthropogenic local emissions released earlier is the maximum (Fig. 8), with ground-level contributions that can reach 30 ppb SE in the municipality of Madrid. However, the greatest contribution during these hours continues to be from the BCs (up to 50–60 ppb at surface level). From 21:00 UTC, the PBL has already decreased to a few hundred meters, the turbulence dwindles, the surface flow towards the SW is reestablished, and the formation of enriched levels of precursors (Fig. 7) and ozone (Fig. 8) in the 1000–2000 m a.s.l. occurs again, in accordance with the regional recirculation processes reported in the literature for this area (Querol et al., 2018; Escudero et al., 2019).

#### 3.3.2 Advective pattern

**Figure 10.**
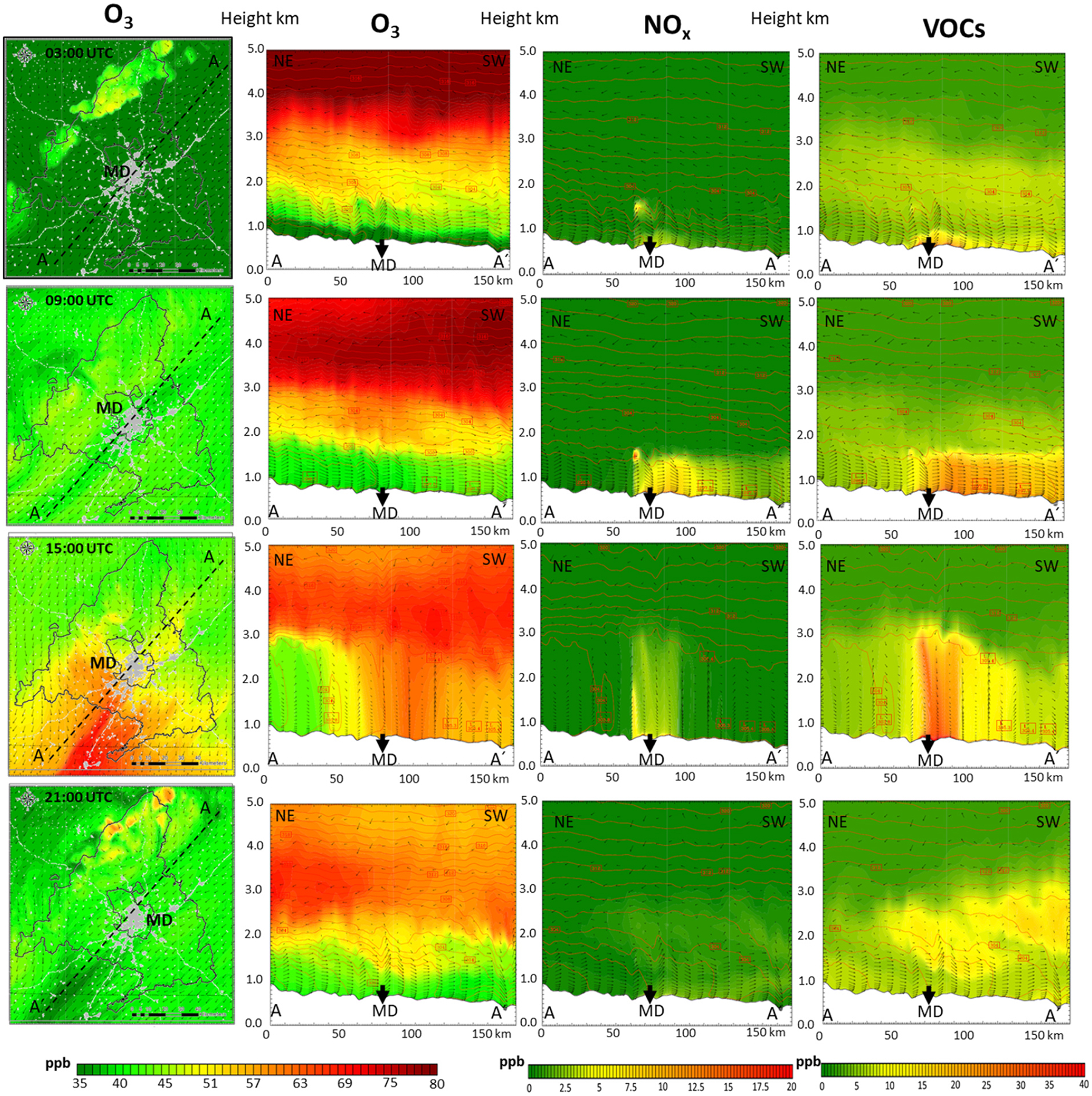
Advective period: hourly evolution during 13 July 2016. From left to right: plan view and NE–SW cross section (up to 5 km height) O_3_ mixing ratios (ppb), NO_*X*_ (ppb), and VOCs (ppb) at the 03:00, 09:00; 15:00, 21:00 UTC hours. MD: Madrid city.

**Figure 11.**
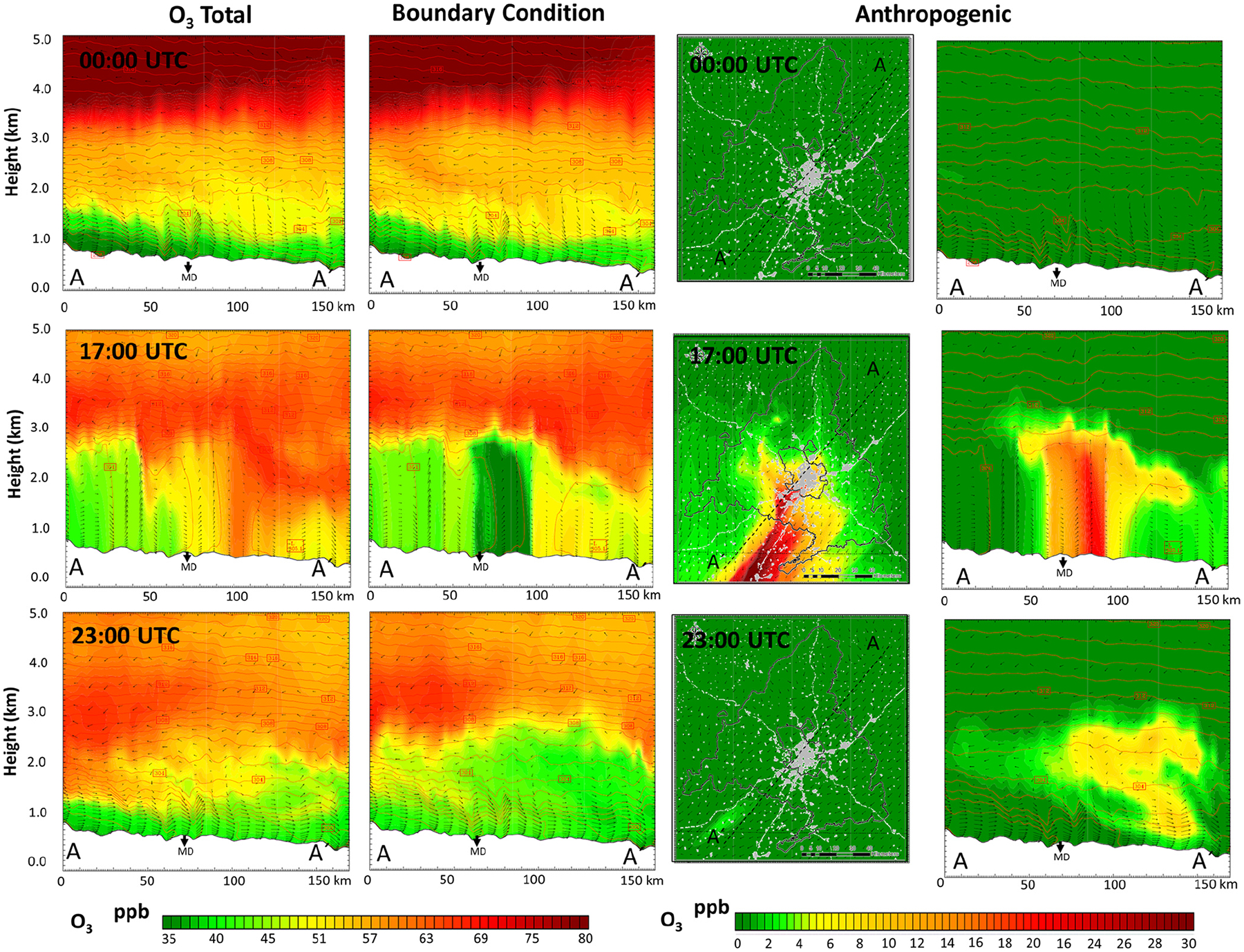
Hourly O_3_ mixing ratios (at 00:00, 17:00; 23:00 UTC) for the NE–SW cross section as well as the contribution of BCs and anthropogenic local emissions on 13 July 2016 (advection). MD = Madrid city.

As an example of an advective pattern, Fig. 10 shows the plan view and the NE–SW cross section of O_3_, NO_*X*_, and VOC levels during 13 July. (Figure S9 shows the SE–NW cross section for day 13, and Figs. S10 and S11 represent the NE–SW cross section and the SE–NW cross section for day 20, respectively.) It can be seen that surface O_3_ levels at 03:00 UTC are around 8 % lower than those of 27 July (accumulation) (in the Madrid region average of 39 and 42 ppb for advective and accumulation conditions, respectively) with maximum values along the Sierra de Guadarrama, where elevated terrain reaches layers rich in O_3_ and precursors from the lower troposphere and from the residual layers formed the day before (Fig. S11). This occurs (also under accumulation conditions) when the PBL height is lower than the maximum height of the Sierra de Guadarrama. However, during advective periods, a stronger stratification of O_3_ is observed during the early hours (03:00–09:00 UTC) due to the existence of more intense wind direction speed vertical gradients (relative to accumulation conditions), perfectly captured by the modeling system (Fig. 11).

At 09:00 UTC, the local O_3_ production downwind of the city is lower than during the accumulation periods (Fig. S12), not only quantitatively but also in terms of the total area affected. This can be explained by the weather conditions (promoting dispersion) and the corresponding lower surface levels of the main precursors (5–8 ppb NO_*X*_ and 15–20 ppb of VOCs on the day 13, compared with 10–15 ppb NO_*X*_ and 30–40 ppb of VOCs during accumulation on day 27). At 15:00 UTC (Fig. 10), the PBL height increases reaching 2500–2800 m altitude (compared with 4000 m on day 27). As the PBL grows, the vertical mixing dominates the wind-driven pollution displacement in the SW direction. Similarly to the dynamics described for accumulation conditions, this allows precursors and fresh O_3_ to ascend and mix existing ozone in higher layers (Figs. 10 and 11). Nonetheless, the vertical mixing is lower during advective patterns, as observed in the ozone soundings (Fig. 9), with the consequent difficulty of the boundary layer to incorporate O_3_ from higher strata (beyond 4000 m a.s.l.) in the central hours of the day. This results in lower O_3_ mixing ratios at surface level under advective conditions, up to 60 ppb SW of Madrid city (Fig. 10). As for the relative importance of local sources, Fig. 11 shows that their contribution can reach nearly 30 ppb, similar to that under accumulation conditions. However, the area affected is clearly associated with the city plume and their contribution averaged over the region is smaller. In fact, our results point out that precursors advected can produce hourly peaks above 30 ppb outside the Madrid region.

### 3.4 Source apportionment assessment at the location of monitoring stations

**Figure 12.**
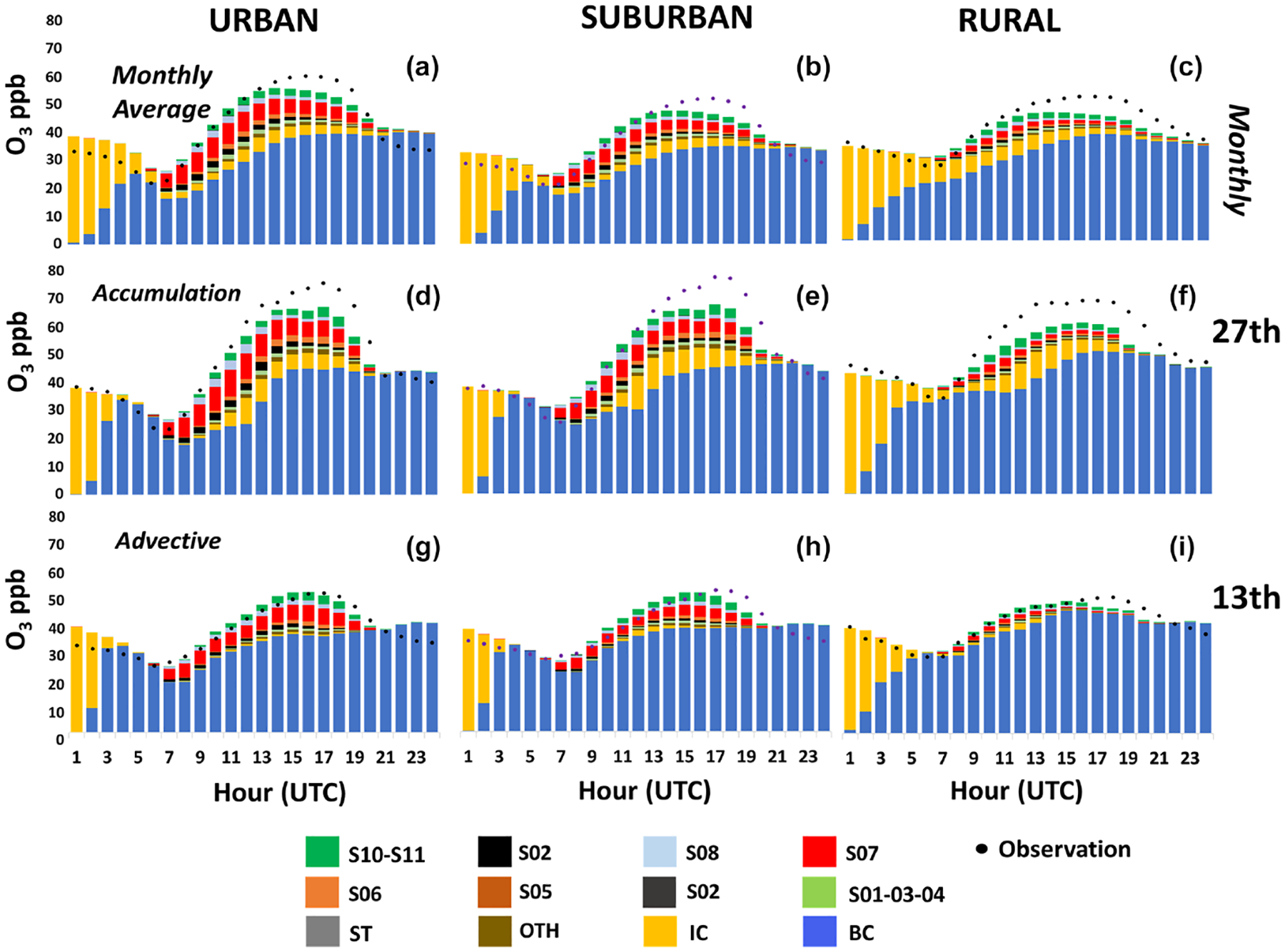
Hourly contribution to ground-level O_3_ for the monthly average (**a–c**) and specifically for accumulation (27 July 2016) and advective (13 July 2016) days (**d–f** and **g–i**, respectively).

A source apportionment assessment has also been carried out at the location of the air quality monitoring stations distributed throughout the simulation domain (Fig. 1) to inform on the contributions of different sources at those points where air quality is routinely monitored. Differences are found depending on the type of station (urban, suburban, or rural) and, consistent with the results discussed in the previous subsection, the type of circulation pattern (advective or accumulation). The results are summarized in Fig. 12. As previously discussed, it shows the contribution of all anthropogenic emission sources (S01–03-04 to S08), biogenic emissions (SNAP10–11) as well as boundary and initial conditions (BCs and ICs), and O_3_ stratospheric transport (ST). Although 100 % of emitting sectors have been tagged, Fig. 12 shows as well the contribution from “others” (OTH). This contribution is typically negligible and relates to minor model interactions between sources and species not considered by the ISAM model. Details are fully explained in the documents provided with the model release (US EPA, 2022).

Urban and suburban monitoring stations have a similar aggregated behavior. During the first hours of the morning, the initial and boundary conditions make up the totality of O_3_ levels until 06:00 UTC approximately. After that time, O_3_ generated from precursors emitted by local sources appears, reaching contributions up to 15 and 12 ppb for urban and suburban locations (28 % and 22 % of the total ozone, respectively) around 12:00 UTC. The road transport (14 %– 10 %) and the residential (2 %–4 %) sectors are those with the highest contributions. The signal of anthropogenic sources is lower in rural monitoring stations. On average, road traffic contributes a maximum of 5 % (5 ppb), the residential sector 2 % (2 ppb), and the use of solvents (VOC emissions) also around 2 % in rural locations.

The results in Fig. 12 demonstrate the persistent relevance of ICs at all locations, but especially in rural locations. Even though the initial conditions contribute to O_3_ levels throughout the day, the maximum values are found in the first hours (00:00–05:00 UTC). As the day evolves, the influence of ICs progressively decreases until they disappear at 21:00 UTC approximately. However, clear differences are found depending on the circulation pattern as illustrated for 27 July (accumulation) and 13 July (advection). According to the model predictions, O_3_ levels are greater during the accumulation period (and are reached slightly earlier), with maxima up to 68 ppb (17:00 UTC) in contrast with 52 ppb under advective conditions. Of note, the model reproduces observed O_3_ temporal patterns quite consistently, but it misses the peak values during accumulation periods, as discussed in Sect. 3.1.

It can be highlighted that the influence of residual layers of the previous day, tracked through the IC tag and observed again during the central hours of the day, is very significant under accumulation conditions (IC contribution of up to 12 ppb, around 18 % of total O_3_), while it is practically missing for advective days. This relates to the enhancement of O_3_ levels from reservoirs aloft, discussed in Sect. 3.3.1, that does not occur under advective conditions. Of note, and consistent with the analysis in Sect. 3.3, we observe that the average contribution from local anthropogenic sources to O_3_ peaks (around 16:00 LT) in urban locations in accumulation periods is higher than that of advective periods. That is true both for absolute levels (18 and 11 ppb, respectively) and relative contributions (32 % and 22 %, respectively). These results point out that the source apportionment under unfavorable circulation patterns significantly differs from that for average or advective conditions and, consistent with previous studies (Lupaşcu et al., 2022; Qu et al., 2023), demonstrate that the influence of local sources is larger for high O_3_ levels under stagnation conditions.

Nonetheless, clear differences are found for individual stations depending on their location relative to the city center and prevailing winds. In the Supplement (Figs. S14–S16) a stratification of the same results by station type and geographical quadrant (Fig. S13), as well as distance to Madrid, is shown. For instance, urban locations within Madrid municipality in the NE direction for 27 July (accumulation) present much higher contributions from local sources than those of urban stations in the NW direction and further away from the metropolitan area (Fig. S15). This variability suggests that the outcome of local measures may differ throughout the region and should be modeled under specific meteorological conditions and assessed specifically for each location of interest.

## 4 Conclusions

A high-resolution chemical-transport model has been used to investigate O_3_ dynamics for a typical summer month (July 2016) in the Madrid region. The model presents an acceptable performance and succeeds in reproducing the phenomena described in previous studies (Querol et al., 2018; Escudero et al., 2019), confirming that O_3_ dynamics are conditioned by regional circulation patterns. Nonetheless, we found that model errors are larger for accumulation days and concentration peaks are underestimated. This may be related to an inadequate performance of the meteorological model under stagnation conditions. A novel implementation of CMAQ-ISAM (Shu et al., 2023) that attributes O_3_-based reaction stoichiometry with all production and destruction reactions involved has been applied to perform a source apportionment of this non-linear, secondary pollutant under specific weather patterns. Our simulation shows that O_3_ levels are dominated by non-local contributions (i.e., boundary conditions), representing around 70 % of mean values across the region. Ozone reservoirs from previous days (labeled as initial conditions in our methodology) in the mid troposphere are also important to build up high O_3_ levels in accumulation episodes, representing the main difference with advective periods. The analysis, however, points out that precursors emitted by local sources play a more important role regarding the highest mixing ratio values, illustrated in this study by the 90th percentile. This suggests that the implementation of emission reduction strategies in the region may be more effective to control O_3_ peaks than average values. This is particularly true under unfavorable, stagnation conditions associated with accumulation patterns when the highest O_3_ values occur. According to our results, up to 35 % of total O_3_ may originate from local sources, giving a theoretical maximum reduction potential of 1 h values of approximately 25 ppb under these conditions. Among local sources, road traffic is the main contributor, accounting for 55 % of local sources. Our results suggest that NO_*X*_ emissions play a more important role than VOC emissions in the photochemical production of ozone. Nonetheless, we found that the use of solvents and other products, a significant source of VOC emissions with high ozone formation potential, can explain up to 20 % of the O_3_ originating from local anthropogenic emissions in some locations. At the same time, our results point out that the contribution of biogenic emissions is lower than that of anthropogenic sources (below 4 % of total O_3_ levels in this period), although they are responsible for 42.4 % of total VOCs in the modeling domain. Emissions from other sectors play a minor role and O_3_ transported from the stratosphere within the model domain is negligible.

We also found significant variations in source apportionment patterns across station types and locations. This implies that high-resolution simulations under specific meteorological conditions should be performed to anticipate the potential outcome on O_3_ levels in different locations of the Madrid region.

Considering these results, future modeling efforts should be oriented to simulate the effect of specific measures, both locally and in cooperation with other administrations, to identify optimal emission abatement strategies. The modeling platform used in this study may be also helpful in assessing sensitivities to different factors, including photochemical regimes or NO_*X*_ and VOC speciation for specific sources. Furthermore, the role of biogenic NO_*X*_ and VOC emissions may be further studied to understand the implications of O_3_ control strategies in the Madrid region.

## Supplementary Material

SI

## Figures and Tables

**Table 1.** Tagged sectors for the O_3_ source apportionment analysis.

Tagged sources	Description	Abbreviation
SNAP01 – SNAP03 – SNAP04	Power generation (S01), Industrial combustion (S03), and Industrial processes without combustion (S04)	S01-03-04
SNAP02	Non-industrial combustion plants	S02
SNAP05	Extraction and distribution of fossil fuels	S05
SNAP06	Use of solvents and other products	S06
SNAP07	Road transport	S07
SNAP08	Other mobile sources and machinery	S08
SNAP09	Waste treatment and disposal	S09
SNAP10 – SNAP11	Agriculture and nature	S10–S11
OTHER	Non-tagged emissions, including online computations (none in this study)	OTH
ICON	Initial conditions	ICs
BCON	Boundary conditions	BCs
PVO3	Stratospheric ozone (potential vorticity)	ST

**Table 2.** Model performance statistics (dimensionless unless noted otherwise) by station type for ground-level O_3_ concentration.

Station type	FAC2	MB (μg m^−3^)	MGE (μg m^−3^)	NMB	NMGE	RMSE (μg m^−3^)	*r*	IOA
Industrial	0.95	7.8	14.5	0.10	0.18	18.7	0.84	0.71
Rural	0.98	−2.9	13.8	−0.03	0.14	18.1	0.76	0.68
Suburban	0.94	2.4	17.15	0.03	0.20	23.3	0.74	0.69
Urban background	0.89	8.3	20.4	0.10	0.25	27.1	0.69	0.65
Urban traffic	0.88	10.8	19.9	0.14	0.25	26.5	0.73	0.65

FAC2: fraction of predictions within a factor of 2; MB: mean bias; MGE: mean gross error; NMB: normalized mean bias, NMGE: normalized mean gross error; RMSE: root mean squared error; *r*: Pearson correlation coefficient; IOA: index of agreement.

## Data Availability

The Community Multiscale Air Quality (CMAQ) and the Integrated Source Apportionment Method (CMAQ-ISAM) are an open-source development project of the US EPA (https://github.com/usepa/cmaq.git, last access: 11 November 2022). The version used in this study (5.3.2) is freely available at https://doi.org/10.5281/zenodo.4081737 (US EPA Office of Research and Development, 2020). Model outputs are available upon request to the authors.

## References

[R1] AEMET: Informe Anual 2016, Ministerio de Agricultura y Pesca, Alimentación y Medio Ambiente, https://www.aemet.es/documentos/es/conocenos/a_que_nos_dedicamos/informes/InformeAnualAEMET_2016_web.pdf (last access: 5 January 2023), 2017.

[R2] AM: Ayuntamiento de Madrid (AM), Inventario de emisiones de contaminantes a la atmósfera en el Término Municipal de Madrid, https://www.madrid.es/UnidadesDescentralizadas/Sostenibilidad/EspeInf/Acci%C3%B3nClim%C3%A1tica/2EstudiosInventarios/4aInventario/ficheros/Inventario%20de%20Emisiones%20Contaminantes%20a%20la%20Atm%C3%B3sfera%20Ayto.%20Madrid%202021.pdf (last access: 7 January 2023), 2021.

[R3] AM: Calidad del Aire Madrid, https://www.madrid.es/UnidadesDescentralizadas/Sostenibilidad/EspeInf/Acci%C3%B3nClim%C3%A1tica/2EstudiosInventarios/4aInventario/ficheros/Inventario%20de%20Emisiones%20Contaminantes%20a%20la%20Atm%C3%B3sfera%20Ayto.%20Madrid%202021.pdf (last access: 7 January 2023), 2022.

[R4] Amann M, Bertok I, Cofala J, Heyes C, Klimont Z, Rafaj P, Schöpp W, and Wagner F: National Emission Ceilings for 2020 based on the 2008 Climate & Energy Package, NEC Scenario Analysis Report No. 6, Final report to the European Commission, International Institute for Applied Systems Analysis (IIASA), Laxenburg, Austria, https://previous.iiasa.ac.at/web/home/research/researchPrograms/air/policy/NEC6-final110708.pdf (last access: 12 June 2023), 2008.

[R5] Appel KW, Pouliot GA, Simon H, Sarwar G, Pye HOT, Napelenok SL, Akhtar F, and Roselle SJ: Evaluation of dust and trace metal estimates from the Community Multiscale Air Quality (CMAQ) model version 5.0, Geosci. Model Dev, 6, 883–899, 10.5194/gmd-6-883-2013, 2013

[R6] Baek BH and Seppanen C: Sparse Matrix Operator Kernel Emissions (SMOKE) Modeling System (Version SMOKE User’s Documentation), 10.5281/zenodo.1421403, 2018.

[R7] Baker K, Woody M, Tonnesen G, Hutzell W, Pye H, Beaver M, Pouliot G, and Pierce T: Contribution of regional-scale fire events to ozone and PM_2.5_ air quality estimated by photochemical modeling approaches, Atmos. Environ, 140, 539–554, 10.1016/j.atmosenv.2016.06.032, 2016.

[R8] Banerjee A, Maycock AC, Archibald AT, Abraham NL, Telford P, Braesicke P, and Pyle JA: Drivers of changes in stratospheric and tropospheric ozone between year 2000 and 2100, Atmos. Chem. Phys, 16, 2727–2746, 10.5194/acp-16-2727-2016, 2016.

[R9] Bates D, Bell G, Burnham C, Hazucha M, Mantha J, Pengelly L, and Silverman F: Short-term effects of ozone on the lung, J. Appl. Physiol, 32, 176–181, 10.1152/jappl.1972.32.2.176, 1972. 5007865

[R10] Bell ML, McDermott A, Zeger SL, Samet JM, and Dominici F: Ozone and short-term mortality in 95 US urban communities, 1987–2000, JAMA, 292, 2372–2378, 10.1001/jama.292.19.2372, 2004. 15547165 PMC3546819

[R11] Borge R, Alexandrov V, Del Vas JJ, Lumbreras J, and Rodríguez E: A comprehensive sensitivity analysis of the WRF model for air quality applications over the Iberian Peninsula, Atmos. Environ, 42, 8560–8574, 10.1016/j.atmosenv.2008.08.032, 2008a.

[R12] Borge R, Lumbreras J, and Rodríguez E: Development of a high-resolution emission inventory for Spain using the SMOKE modelling system: a case study for the years 2000 and 2010, Environ. Modell. Softw, 23, 1026–1044, 10.1016/j.envsoft.2007.11.002, 2008b.

[R13] Borge R, López J, Lumbreras J, Narros A, and Rodríguez E: Influence of boundary conditions on CMAQ simulations over the Iberian Peninsula, Atmos. Environ, 44, 2681–2695, 10.1016/j.atmosenv.2010.04.044, 2010.

[R14] Borge R, Lumbreras J, Pérez J, de la Paz D, Vedrenne M, de Andrés JM, and Rodríguez ME: Emission inventories and modeling requirements for the development of air quality plans. Application to Madrid (Spain), Sci. Total Environ, 466–467, 809–819, 10.1016/j.scitotenv.2013.07.093, 2014. 23973547

[R15] Borge R, Santiago JL, de la Paz D, Martín F, Domingo J, Valdes C, Sanchez B, Rivas E, Rozas MT, Lázaro S, Perez J, and Fernandez A: Application of a short term air quality action plan in Madrid (Spain) under a high-pollution episode – Part II: Assessment from multi-scale modelling, Sci. Total Environ, 635, 1574–1584, 10.1016/j.scitotenv.2018.04.323, 2018. 29739658

[R16] Borge R, Requia WJ, Yagüe C, Jhun I, and Koutrakis P: Impact of weather changes on air quality and related mortality in Spain over a 25 year period [1993–2017], Environ. Int, 133, 105272, 10.1016/j.envint.2019.105272, 2019. 31675571

[R17] Borge R, de la Paz D, Sarwar G, and Napelenok S: Comparison of source apportionment methods using CMAQ for the Madrid region, in: 21st Annual CMAS Conference, Chapel Hill, NC, 17–19 October 2022, https://www.cmascenter.org/conference/2022/slides/0920am_ComparisonSourceApportionment_RBorge.pptx (last access: 11 February 2023), 2022.

[R18] Brodin M, Helmig D, and Oltmans S: Seasonal ozone behavior along an elevation gradient in the Colorado Front Range Mountains, Atmos. Environ, 44, 5305–5315, 10.1016/j.atmosenv.2010.06.033, 2010.

[R19] Brook JR, Makar PA, Sills DML, Hayden KL, and McLaren R: Exploring the nature of air quality over southwestern Ontario: main findings from the Border Air Quality and Meteorology Study, Atmos. Chem. Phys, 13, 10461–10482, 10.5194/acp-13-10461-2013, 2013.

[R20] Butler T, Lupascu A, Coates J, and Zhu S: TOAST 1.0: Tropospheric Ozone Attribution of Sources with Tagging for CESM 1.2.2, Geosci. Model Dev, 11, 2825–2840, 10.5194/gmd-11-2825-2018, 2018.

[R21] Butler T, Lupascu A, and Nalam A: Attribution of ground-level ozone to anthropogenic and natural sources of nitrogen oxides and reactive carbon in a global chemical transport model, Atmos. Chem. Phys, 20, 10707–10731, 10.5194/acp-20-10707-2020, 2020.

[R22] Byun D and Schere KL: Review of the governing equations, computational algorithms, and other components of the Models-3 Community Multiscale Air Quality (CMAQ) modeling system, Appl. Mech. Rev, 59, 51–77, 10.1115/1.2128636, 2006.

[R23] Cao J, Qiu X, Liu Y, Yan X, Gao J, and Peng L: Identifying the dominant driver of elevated surface ozone concentration in North China plain during summertime 2012–2017, Environ. Pollut, 300, 118912, 10.1016/j.envpol.2022.118912, 2022. 35092729

[R24] Carnero JAA, Bolívar JP, and Benito A: Surface ozone measurements in the southwest of the Iberian Peninsula (Huelva, Spain), Environ. Sci. Pollut. R, 17, 355–368, 10.1007/s11356-008-0098-9, 2009. 19153676

[R25] Carter WPL: Updated maximum incremental reactivity scale and hydrocarbon bin reactivities for regulatory applications, California Air Resources Board Contract, vol. 339, https://ww2.arb.ca.gov/sites/default/files/barcu/regact/2009/mir2009/mir10.pdf (last access: 7 February 2023), 2009.

[R26] Carter WPL and Atkinson R: Computer modeling study of incremental hydrocarbon reactivity, Environ. Sci. Technol, 23, 864–880, 10.1021/es00065a017, 1989. 19995044

[R27] Ching J and Byun D: Introduction to the Models-3 framework and the Community Multiscale Air Quality model (CMAQ), Science Algorithms of the EPA Models-3 Community Multiscale Air Quality (CMAQ) Modeling System, https://www.cmascenter.org/cmaq/science_documentation/pdf/ch01.pdf (last access: 21 November 2022), 1999.

[R28] Ciccioli P, Silibello C, Finardi S, Pepe N, Ciccioli P, Rapparini F, Neri L, Fares S, Brilli F, Mircea M, Magliulo E, and Baraldi R: The potential impact of biogenic volatile organic compounds (BVOCs) from terrestrial vegetation on a Mediterranean area using two different emission models, Agr. Forest Meteorol, 328, 109255, 10.1016/j.agrformet.2022.109255, 2023.

[R29] CM: Inventario de emisiones a la atmósfera en la Comunidad de Madrid. Años 1990–2018, Comunidad de Madrid, Dirección General de Sostenibilidad y Cambio Climático, https://www.comunidad.madrid/sites/default/files/doc/medio-ambiente/documento_de_sintesis_inventario_de_emisiones_comunidad_de_madrid.pdf (last access: 14 August 2022), 2021.

[R30] Coggon MM, Gkatzelis GI, McDonald BC, Gilman JB, Schwantes RH, Abuhassan N, and Warneke C: Volatile chemical product emissions enhance ozone and modulate urban chemistry, P. Natl. Acad. Sci. USA, 118, e2026653118, 10.1073/pnas.2026653118, 2021. PMC8364211 34341119

[R31] Cohan DS and Napelenok SL: Air quality response modeling for decision support, Atmosphere, 2, 407–425, 10.3390/atmos2030407, 2011.

[R32] Collet S, Kidokoro T, Karamchandani P, Jung J, and Shah T: Future year ozone source attribution modeling study using CMAQ-ISAM, J. Air Waste Manage, 68, 1239–1247, 10.1080/10962247.2018.1496954, 2018. 29999477

[R33] De Andrés JM, Borge R, De La Paz D, Lumbreras J, and Rodríguez E: Implementation of a module for risk of ozone impacts assessment to vegetation in the Integrated Assessment Modelling system for the Iberian Peninsula. Evaluation for wheat and Holm oak, Environ. Pollut, 165, 25–37, 10.1016/j.envpol.2012.01.048, 2012. 22398018

[R34] de la Paz D, Borge R, and Martilli A: Assessment of a high resolution annual WRF-BEP/CMAQ simulation for the urban area of Madrid (Spain), Atmos. Environ, 144, 282–296, 10.1016/j.atmosenv.2016.08.082, 2016.

[R35] de la Paz D, Borge R, Perez J, and de Andrés JM: Contributions to summer ground-level O3 in the Madrid Region, Proceedings of Abstracts of the 12th International Conference on Air Quality Science and Application, Thessaloniki, Greece, 18–22 May 2020, 153, 10.18745/PB.22217, 2020.

[R36] Dunker AM, Koo B, and Yarwood G: Ozone sensitivity to isoprene chemistry and emissions and anthropogenic emissions in central California, Atmos. Environ, 145, 326–337, 10.1016/j.atmosenv.2016.09.048, 2016.

[R37] EEA: EMEP/EEA air pollutant emission inventory guidebook 2019. Technical guidance to prepare national emission inventories, EEA Report no. 13/2019, European Environmental Agency (EEA), 10.2800/293657, https://www.eea.europa.eu/publications/emep-eea-guidebook-2019 (last access: 22 January 2023), 2019.

[R38] EEA: Air quality in europe 2020 report, European Environment Agency, 10.2800/786656, 2020.

[R39] EEA: European Union emission inventory report 1990–2020 under the UNECE Air Convention European Environment Agency, Publications Office of the European Union, Luxembourg, 10.2800/928370, 2022.

[R40] Emery C, Jung J, Koo B, and Yarwood G: Final Report, Improvements to CAMx Snow Cover Treatments and Carbon Bond Chemical Mechanism for Winter Ozone, Tech. rep., Ramboll Environ, https://www.camx.com/files/emaq4-07_task7_techmemo_r1_1aug16.pdf (last access: 22 March 2023), 2015.

[R41] Escudero M, Segers A, Kranenburg R, Querol X, Alastuey A, Borge R, de la Paz D, Gangoiti G, and Schaap M: Analysis of summer O_3_ in the Madrid air basin with the LOTOS-EUROS chemical transport model, Atmos. Chem. Phys, 19, 14211–14232, 10.5194/acp-19-14211-2019, 2019.

[R42] European Environment Agency, Guerreiro C, Colette A, Leeuw F, and González Ortiz A: Air quality in Europe 2018 report, European Environmental Agency (EEA), Publications Office, 10.2800/777411, 2018.

[R43] García R, Prieto L, Díaz J, Hernández E, and del Teso T: Synoptic conditions leading to extremely high temperatures in Madrid, Ann. Geophys, 20, 237–245, 10.5194/angeo-20-237-2002, 2002.

[R44] Garrido-Pérez JM, Ordóñez C, García-Herrera R, and Schnell JL: The differing impact of air stagnation on near-surface ozone across Europe, EGU General Assembly 2020, Online, 4–8 May 2020, EGU2020–9213, 10.5194/egusphereegu2020-9213, 2020.

[R45] Gaudel A, Cooper O, Ancellet G, Barret B, Boynard A, Burrows J, Clerbaux C, Coheur P-F, Cuesta J, and Cuevas Agulló E: Tropospheric Ozone Assessment Report: Presentday distribution and trends of tropospheric ozone relevant to climate and global atmospheric chemistry model evaluation, Elem. Sci. Anth, 6, 39, 10.1525/elementa.291, 2018.

[R46] Goodman JE, Zu K, Loftus CT, Lynch HN, Prueitt RL, Mohar I, Shubin SP, and Sax SN: Short-term ozone exposure and asthma severity: Weight-of-evidence analysis, Environ. Res, 160, 391–397, 10.1016/j.envres.2017.10.018, 2018. 29059621

[R47] Granados-Muñoz MJ and Leblanc T: Tropospheric ozone seasonal and long-term variability as seen by lidar and surface measurements at the JPL-Table Mountain Facility, California, Atmos. Chem. Phys, 16, 9299–9319, 10.5194/acp-16-9299-2016, 2016.

[R48] Grewe V, Tsati E, Mertens M, Frömming C, and Jöckel P: Contribution of emissions to concentrations: the TAGGING 1.0 submodel based on the Modular Earth Submodel System (MESSy 2.52), Geosci. Model Dev, 10, 2615–2633, 10.5194/gmd-10-2615-2017, 2017.

[R49] Guenther A, Karl T, Harley P, Wiedinmyer C, Palmer PI, and Geron C: Estimates of global terrestrial isoprene emissions using MEGAN (Model of Emissions of Gases and Aerosols from Nature), Atmos. Chem. Phys, 6, 3181–3210, 10.5194/acp-6-3181-2006, 2006.

[R50] Guenther AB, Jiang X, Heald CL, Sakulyanontvittaya T, Duhl T, Emmons LK, and Wang X: The Model of Emissions of Gases and Aerosols from Nature version 2.1 (MEGAN2.1): an extended and updated framework for modeling biogenic emissions, Geosci. Model Dev, 5, 1471–1492, 10.5194/gmd-5-1471-2012, 2012.

[R51] Han X, Zhu L, Wang S, Meng X, Zhang M, and Hu J: Modeling study of impacts on surface ozone of regional transport and emissions reductions over North China Plain in summer 2015, Atmos. Chem. Phys, 18, 12207–12221, 10.5194/acp-18-12207-2018, 2018.

[R52] Harmens H, Mills G, Hayes F, and Norris D: Air pollution and vegetation: ICP Vegetation annual report 2010/2011, ISBN 978–1-906698–26-3, 2011.

[R53] Hsu J, Prather MJ, and Wild O: Diagnosing the stratosphere-to-troposphere flux of ozone in a chemistry transport model, J. Geophys. Res.-Atmos, 110, D19305, 10.1029/2005JD006045, 2005.

[R54] Hsu Y, Strait R, Roe S, and Holoman D: SPECIATE 4.0 Speciation database development documentation: Final Report, EPA/600/R-06/161, US Environmental Protection Agency, Office of Research and and Development U. S. Environmental Protection Agency, Research Triangle Park, NC 27711, https://cfpub.epa.gov/si/si_public_file_download.cfm?p_download_id=459904&Lab=NRMRL (last access: 21 March 2023), 2006.

[R55] IPCC: Climate Change 2007: The Physical Science Basis. Contribution of Working Group I to the Fourth Assessment Report of the Intergovernmental Panel on Climate Change, edited by: Solomon S, Qin D, Manning M, Chen Z, Marquis M, Averyt KB, Tignor M, and Miller HL, Cambridge University Press, Cambridge, United Kingdom and New York, NY, USA, 996 pp., ISBN 978–0-521–88009-1, https://www.ipcc.ch/site/assets/uploads/2018/05/ar4_wg1_full_report-1.pdf (last access: 21 March 2023), 2007.

[R56] IPCC: Climate Change 2014: Mitigation of Climate Change. Contribution of Working Group III to the Fifth Assessment Report of the Intergovernmental Panel on Climate Change, edited by: Edenhofer O, Pichs-Madruga R, Sokona Y, Farahani E, Kadner S, Seyboth K, Adler A, Baum I, Brunner S, Eickemeier P, Kriemann B, Savolainen J, Schlömer S, von Stechow C, Zwickel T, and Minx JC, Cambridge University Press, Cambridge, United Kingdom and New York, NY, USA, ISBN 978–1-107–05821-7, https://www.ipcc.ch/site/assets/uploads/2018/02/ipcc_wg3_ar5_full.pdf (last access: 21 March 2023), 2014.

[R57] Jenkin ME and Clemitshaw KC: Ozone and other secondary photochemical pollutants: chemical processes governing their formation in the planetary boundary layer, Atmos. Environ, 34, 2499–2527, 10.1016/S1352-2310(99)00478-1, 2000.

[R58] Jerrett M, Burnett RT, Pope CA III, Ito K, Thurston G, Krewski D, Shi Y, Calle E, and Thun M: Long-term ozone exposure and mortality, New Engl. J. Med, 360, 1085–1095, https://www.nejm.org/doi/full/10.1056/nejmoa0803894 (last access: 24 March 2023), 2009. 19279340 10.1056/NEJMoa0803894 PMC4105969

[R59] Jhun I, Coull BA, Zanobetti A, and Koutrakis P: The impact of nitrogen oxides concentration decreases on ozone trends in the USA, Air Qual. Atmos. Hlth, 8, 283–292, 10.1007/s11869-014-0279-2, 2015. PMC4988408 27547271

[R60] Jiang J, Aksoyoglu S, Ciarelli G, Oikonomakis E, El-Haddad I, Canonaco F, O’Dowd C, Ovadnevaite J, Minguillón MC, Baltensperger U, and Prévôt ASH: Effects of two different biogenic emission models on modelled ozone and aerosol concentrations in Europe, Atmos. Chem. Phys, 19, 3747–3768, 10.5194/acp-19-3747-2019, 2019.

[R61] Jung D, de la Paz D, Notario A, and Borge R: Analysis of emissions-driven changes in the oxidation capacity of the atmosphere in Europe, Sci. Total Environ, 827, 154126, 10.1016/j.scitotenv.2022.154126, 2022. 35219666

[R62] Jung D, Soler R, de la Paz D, Notario A, Muñoz A, Ródenas M, Vera T, Borrás E, and Borge R: Oxidation capacity changes in the atmosphere of large urban areas in Europe: Modelling and experimental campaigns in atmospheric simulation chambers, Chemosphere, 341, 139919, 10.1016/j.chemosphere.2023.139919, 2023. 37611775

[R63] Karamchandani P, Long Y, Pirovano G, Balzarini A, and Yarwood G: Source-sector contributions to European ozone and fine PM in 2010 using AQMEII modeling data, Atmos. Chem. Phys, 17, 5643–5664, 10.5194/acp-17-5643-2017, 2017.

[R64] Kwok RHF, Napelenok SL, and Baker KR: Implementation and evaluation of PM_2.5_ source contribution analysis in a photochemical model, Atmos. Environ, 80, 398–407, 10.1016/j.atmosenv.2013.08.017, 2013.

[R65] Kwok RHF, Baker KR, Napelenok SL, and Tonnesen GS: Photochemical grid model implementation and application of VOC, NO_*x*_, and O_3_ source apportionment, Geosci. Model Dev, 8, 99–114, 10.5194/gmd-8-99-2015, 2015.

[R66] Li X, Qin M, Li L, Gong K, Shen H, Li J, and Hu J: Examining the implications of photochemical indicators for O_3_–NO_*x*_–VOC sensitivity and control strategies: a case study in the Yangtze River Delta (YRD), China, Atmos. Chem. Phys, 22, 14799–14811, 10.5194/acp-22-14799-2022, 2022.

[R67] Logan JA: Tropospheric ozone: Seasonal behavior, trends, and anthropogenic influence, J. Geophys. Res.-Atmos, 90, 10463–10482, 10.1029/JD090iD06p10463, 1985.

[R68] Lu X, Ye X, Zhou M, Zhao Y, Weng H, Kong H, Li K, Gao M, Zheng B, and Lin J: The underappreciated role of agricultural soil nitrogen oxide emissions in ozone pollution regulation in North China, Nat. Commun, 12, 5021, 10.1038/s41467-021-25147-9, 2021. 34408153 PMC8373933

[R69] Lupaşcu A, Otero N, Minkos A, and Butler T: Attribution of surface ozone to NO_*x*_ and volatile organic compound sources during two different high ozone events, Atmos. Chem. Phys, 22, 11675–11699, 10.5194/acp-22-11675-2022, 2022.

[R70] Massagué J, Escudero M, Alastuey A, Mantilla E, Monfort E, Gangoiti G, García-Pando CP, and Querol X: Spatiotemporal variations of tropospheric ozone in Spain (2008–2019), Environ. Int, 176, 107961, 10.1016/j.envint.2023.107961, 2023. 37216837

[R71] Mathur R, Xing J, Gilliam R, Sarwar G, Hogrefe C, Pleim J, Pouliot G, Roselle S, Spero TL, Wong DC, and Young J: Extending the Community Multiscale Air Quality (CMAQ) modeling system to hemispheric scales: overview of process considerations and initial applications, Atmos. Chem. Phys, 17, 12449–12474, 10.5194/acp-17-12449-2017, 2017. 29681922 PMC5907506

[R72] Meng Y, Song J, Zeng L, Zhang Y, Zhao Y, Liu X, Guo H, Zhong L, Ou Y, Zhou Y, Zhang T, Yue D, and Lai S: Ambient volatile organic compounds at a receptor site in the Pearl River Delta region: Variations, source apportionment and effects on ozone formation, J. Environ. Sci, 111, 104–117, 10.1016/j.jes.2021.02.024, 2022. 34949340

[R73] Meul S, Langematz U, Kröger P, Oberländer-Hayn S, and Jöckel P: Future changes in the stratosphere-to-troposphere ozone mass flux and the contribution from climate change and ozone recovery, Atmos. Chem. Phys, 18, 7721–7738, 10.5194/acp-18-7721-2018, 2018.

[R74] Millán MM, Mantilla E, Salvador R, Carratalá A, Sanz MJ, Alonso L, Gangoiti G, and Navazo M: Ozone cycles in the western Mediterranean basin: interpretation of monitoring data in complex coastal terrain, J. Appl. Meteorol, 39, 487–508, 10.1175/1520-0450(2000)039<0487:OCITWM>2.0.CO;2, 2000.

[R75] Mills G, Pleijel H, Braun S, Büker P, Bermejo V, Calvo E, Danielsson H, Emberson L, Fernández IG, and Grünhage L: New stomatal flux-based critical levels for ozone effects on vegetation, Atmos. Environ, 45, 5064–5068, 10.1016/j.atmosenv.2011.06.009, 2011.

[R76] MMA: Inventario Nacional de contaminantes atmosféricos, https://unfccc.int/resource/podcast/nir/ES_NIR_UNFCCC_2018.pdf (last access: 24 March 2023), 2018.

[R77] Monks PS, Archibald AT, Colette A, Cooper O, Coyle M, Derwent R, Fowler D, Granier C, Law KS, Mills GE, Stevenson DS, Tarasova O, Thouret V, von Schneidemesser E, Sommariva R, Wild O, and Williams ML: Tropospheric ozone and its precursors from the urban to the global scale from air quality to short-lived climate forcer, Atmos. Chem. Phys, 15, 8889–8973, 10.5194/acp-15-8889-2015, 2015.

[R78] Napelenok S: Description of the ISAM Chemistry Method, https://github.com/USEPA/CMAQ/blob/main/DOCS/Users_Guide/CMAQ_UG_ch11_ISAM.md (last access: 24 March 2023), 2020.

[R79] Nguyen D-H, Lin C, Vu C-T, Cheruiyot NK, Nguyen MK, Le TH, Lukkhasorn W, and Bui X-T: Tropospheric ozone and NO_*X*_: a review of worldwide variation and meteorological influences, Environmental Technology & Innovation, 28, 102809, 10.1016/j.eti.2022.102809, 2022.

[R80] Oliveira K, Guevara M, Jorba O, Querol X, and García-Pando CP: A new NMVOC speciated inventory for a reactivity-based approach to support ozone control strategies in Spain, Sci. Total Environ, 867, 161449, 10.1016/j.scitotenv.2023.161449, 2023. 36623647 PMC9938404

[R81] Otte TL and Pleim JE: The Meteorology-Chemistry Interface Processor (MCIP) for the CMAQ modeling system: updates through MCIPv3.4.1, Geosci. Model Dev, 3, 243–256, 10.5194/gmd-3-243-2010, 2010.

[R82] Paoletti E, De Marco A, Beddows DC, Harrison RM, and Manning WJ: Ozone levels in European and USA cities are increasing more than at rural sites, while peak values are decreasing, Environ. Pollut, 192, 295–299, 10.1016/j.envpol.2014.04.040, 2014. 24906864

[R83] Pay MT, Gangoiti G, Guevara M, Napelenok S, Querol X, Jorba O, and Pérez García-Pando C: Ozone source apportionment during peak summer events over southwestern Europe, Atmos. Chem. Phys, 19, 5467–5494, 10.5194/acp-19-5467-2019, 2019. 33424952 PMC7788066

[R84] Plaza J, Pujadas M, and Artíñano B: Formation and transport of the Madrid ozone plume, J. Air Waste Manage, 47, 766–774, 10.1080/10473289.1997.10463938, 1997.

[R85] Poupkou A, Giannaros T, Markakis K, Kioutsioukis I, Curci G, Melas D, and Zerefos C: A model for European Biogenic Volatile Organic Compound emissions: Software development and first validation, Environ. Modell. Softw, 25, 1845–1856, 10.1016/j.envsoft.2010.05.004, 2010.

[R86] Qu K, Wang X, Cai X, Yan Y, Jin X, Vrekoussis M, Kanakidou M, Brasseur GP, Shen J, Xiao T, Zeng L, and Zhang Y: Rethinking the role of transport and photochemistry in regional ozone pollution: insights from ozone concentration and mass budgets, Atmos. Chem. Phys, 23, 7653–7671, 10.5194/acp-23-7653-2023, 2023.

[R87] Qu Z, Wu D, Henze DK, Li Y, Sonenberg M, and Mao F: Transboundary transport of ozone pollution to a US border region: A case study of Yuma, Environ. Pollut, 273, 116421, 10.1016/j.envpol.2020.116421, 2021. 33460873

[R88] Quaassdorff C, Borge R, Pérez J, Lumbreras J, de la Paz D, and de Andrés JM: Microscale traffic simulation and emission estimation in a heavily trafficked roundabout in Madrid (Spain), Sci. Total Environ, 566, 416–427, 10.1016/j.scitotenv.2016.05.051, 2016. 27232968

[R89] Querol X, Alastuey A, Pandolfi M, Reche C, Pérez N, Minguillón MC, Moreno T, Viana M, Escudero M, and Orio A: 2001–2012 trends on air quality in Spain, Sci. Total Environ, 490, 957–969, 10.1016/j.scitotenv.2014.05.074, 2014. 24911774

[R90] Querol X, Alastuey A, Reche C, Orio A, Pallares M, Reina F, Dieguez J, Mantilla E, Escudero M, and Alonso L: On the origin of the highest ozone episodes in Spain, Sci. Total Environ, 572, 379–389, 10.1016/j.scitotenv.2016.07.193, 2016. 27509076

[R91] Querol X, Gangoiti G, Mantilla E, Alastuey A, Minguillón MC, Amato F, Reche C, Viana M, Moreno T, Karanasiou A, Rivas I, Pérez N, Ripoll A, Brines M, Ealo M, Pandolfi M, Lee H-K, Eun H-R, Park Y-H, Escudero M, Beddows D, Harrison RM, Bertrand A, Marchand N, Lyasota A, Codina B, Olid M, Udina M, Jiménez-Esteve B, Soler MR, Alonso L, Millán M, and Ahn K-H: Phenomenology of high-ozone episodes in NE Spain, Atmos. Chem. Phys, 17, 2817–2838, 10.5194/acp-17-2817-2017, 2017.

[R92] Querol X, Alastuey A, Gangoiti G, Perez N, Lee HK, Eun HR, Park Y, Mantilla E, Escudero M, Titos G, Alonso L, Temime-Roussel B, Marchand N, Moreta JR, Revuelta MA, Salvador P, Artíñano B, García dos Santos S, Anguas M, Notario A, Saiz-Lopez A, Harrison RM, Millán M, and Ahn K-H: Phenomenology of summer ozone episodes over the Madrid Metropolitan Area, central Spain, Atmos. Chem. Phys, 18, 6511–6533, 10.5194/acp-18-6511-2018, 2018.

[R93] Reche C, Moreno T, Amato F, Pandolfi M, Pérez J, de La Paz D, Diaz E, Gómez-Moreno F, Pujadas M, and Artíñano B: Spatio-temporal patterns of high summer ozone events in the Madrid Basin, Central Spain, Atmos. Environ, 185, 207–220, 10.1016/j.atmosenv.2018.05.002, 2018.

[R94] Saiz-Lopez A, Borge R, Notario A, Adame JA, de la Paz D, Querol X, Artíñano B, Gómez-Moreno FJ, and Cuevas CA: Unexpected increase in the oxidation capacity of the urban atmosphere of Madrid, Spain, Sci. Rep, 7, 45956, 10.1038/srep45956, 2017. 28397785 PMC5387723

[R95] San José R, Stohl A, Karatzas K, Bohler T, James P, and Pérez JL: A modelling study of an extraordinary night time ozone episode over Madrid domain, Environ. Modell. Softw, 20, 587–593, 10.1016/j.envsoft.2004.03.009, 2005.

[R96] Sartelet KN, Couvidat F, Seigneur C, and Roustan Y: Impact of biogenic emissions on air quality over Europe and North America, Atmos. Environ, 53, 131–141, 10.1016/j.atmosenv.2011.10.046, 2012.

[R97] Sarwar G, Simon H, Bhave P, and Yarwood G: Examining the impact of heterogeneous nitryl chloride production on air quality across the United States, Atmos. Chem. Phys, 12, 6455–6473, 10.5194/acp-12-6455-2012, 2012.

[R98] Seinfeld JH and Pandis SN: Atmospheric chemistry and physics: from air pollution to climate change, John Wiley & Sons, ISBN 978–1-118–94740-1, 2016.

[R99] Seltzer KM, Shindell DT, and Malley CS: Measurement-based assessment of health burdens from long-term ozone exposure in the United States, Europe, and China, Environ. Res. Lett, 13, 104018, 10.1088/1748-9326/aae29d, 2018.

[R100] Shu Q, Napelenok SL, Hutzell WT, Baker KR, Henderson BH, Murphy BN, and Hogrefe C: Comparison of ozone formation attribution techniques in the northeastern United States, Geosci. Model Dev, 16, 2303–2322, 10.5194/gmd-16-2303-2023, 2023. PMC11694848 39748926

[R101] Sicard P, Agathokleous E, Anenberg SC, De Marco A, Paoletti E, and Calatayud V: Trends in urban air pollution over the last two decades: A global perspective, Sci. Total Environ, 858, 160064, 10.1016/j.scitotenv.2022.160064, 2023. 36356738

[R102] Sillman S: The use of NO_*y*_, H_2_O_2_, and HNO_3_ as indicators for ozone-NO_*X*_-hydrocarbon sensitivity in urban locations, J. Geophys. Res, 100, 14175–14188, 10.1029/94JD02953, 1995.

[R103] Simon H, Valin LC, Baker KR, Henderson BH, Crawford JH, Pusede SE, Kelly JT, Foley KM, Chris Owen R, and Cohen RC: Characterizing CO and NO_*y*_ sources and relative ambient ratios in the Baltimore area using ambient measurements and source attribution modeling, J. Geophys. Res.-Atmos, 123, 3304–3320, 10.1002/2017JD027688, 2018. 35958736 PMC9364951

[R104] Simpson D: Biogenic emissions in Europe: 2. Implications for ozone control strategies, J. Geophys. Res, 100, 22891–22906, 10.1029/95JD01878, 1995.

[R105] Sitch S, Cox P, Collins W, and Huntingford C: Indirect radiative forcing of climate change through ozone effects on the land-carbon sink, Nature, 448, 791, 10.1038/nature06059, 2007. 17653194

[R106] Skamarock WC and Klemp JB: A time-split nonhydrostatic atmospheric model for weather research and forecasting applications, J. Comput. Phys, 227, 3465–3485, 10.1016/j.jcp.2007.01.037, 2008.

[R107] Stevenson D, Dentener F, Schultz M, Ellingsen K, Van Noije T, Wild O, Zeng G, Amann M, Atherton C, and Bell N: Multimodel ensemble simulations of present-day and near-future tropospheric ozone, J. Geophys. Res.-Atmos, 111, D08301, 10.1029/2005JD006338, 2006.

[R108] Stocker TF, Qin D, Plattner G-K, Tignor M, Allen SK, Boschung J, Nauels A, Xia Y, Bex V, and Midgley PM: Climate change 2013: The physical science basis, https://www.ipcc.ch/site/assets/uploads/2018/02/WG1AR5_all_final.pdf (last access: 1 April 2023), 2013.

[R109] Tagaris E, Sotiropoulou REP, Gounaris N, Andronopoulos S, and Vlachogiannis D: Impact of biogenic emissions on ozone and fine particles over Europe: Comparing effects of temperature increase and a potential anthropogenic NO_*X*_ emissions abatement strategy, Atmos. Environ, 98, 214–223, 10.1016/j.atmosenv.2014.08.056, 2014.

[R110] Thunis P, Clappier A, Tarrasón L, Cuvelier C, Monteiro A, Pisoni E, Wesseling J, Belis C, Pirovano G, and Janssen S: Source apportionment to support air quality planning: Strengths and weaknesses of existing approaches, Environ. Int, 130, 104825, 10.1016/j.envint.2019.05.019, 2019. 31226558 PMC6686078

[R111] UNC: SMOKE’s V365 User’s Manual, University of North Carolina at Chapel Hill, https://www.cmascenter.org/smoke/documentation/3.6.5/manual_smokev365.pdf (last access: 22 November 2022), 2015.

[R112] U.S. EPA: Community Multiscale Air Quality (CMAQ) model v5.4 User Guide, Office of Research and Development, U.S. EPA, https://github.com/USEPA/CMAQ/tree/5.4/DOCS/Users_Guide (last access: 22 January 2021), 2022.

[R113] US EPA Office of Research and Development: CMAQ (5.3.2), Zenodo [code], 10.5281/zenodo.4081737, 2020.

[R114] Valverde V, Pay MT, and Baldasano JM: Ozone attributed to Madrid and Barcelona on-road transport emissions: Characterization of plume dynamics over the Iberian Peninsula, Sci. Total Environ, 543, 670–682, 10.1016/j.scitotenv.2015.11.070, 2016. 26615485

[R115] Visser AJ, Boersma KF, Ganzeveld LN, and Krol MC: European NO_*x*_ emissions in WRF-Chem derived from OMI: impacts on summertime surface ozone, Atmos. Chem. Phys, 19, 11821–11841, 10.5194/acp-19-11821-2019, 2019.

[R116] Wang P, Schade G, Estes M, and Ying Q: Improved MEGAN predictions of biogenic isoprene in the contiguous United States, Atmos. Environ, 148, 337–351, 10.1016/j.atmosenv.2016.11.006, 2017.

[R117] Weng H, Lin J, Martin R, Millet BM, Jaeglé L, Ridley D, Keller C, Li C, Du M, and Meng J: Global high-resolution emissions of soil NO_*X*_, sea salt aerosols, and biogenic volatile organic compounds, Sci. Data, 7, 148, 10.1038/s41597-020-0488-5, 2020. 32433468 PMC7239948

[R118] Whitten GZ, Heo G, Kimura Y, McDonald-Buller E, Allen DT, Carter WP, and Yarwood G: A new condensed toluene mechanism for Carbon Bond: CB05-TU, Atmos. Environ, 44, 5346–5355, 10.1016/j.atmosenv.2009.12.029, 2010.

[R119] WHO: WHO global air quality guidelines: particulate matter (PM_2.5_ and PM_10_), ozone, nitrogen dioxide, sulfur dioxide and carbon monoxide: executive summary, ISBN 9789240034228, 2021. 34662007

[R120] Xu J, Ma JZ, Zhang XL, Xu XB, Xu XF, Lin WL, Wang Y, Meng W, and Ma ZQ: Measurements of ozone and its precursors in Beijing during summertime: impact of urban plumes on ozone pollution in downwind rural areas, Atmos. Chem. Phys, 11, 12241–12252, 10.5194/acp-11-12241-2011, 2011.

[R121] Yarwood G, Jung J, Whitten G, Heo G, Mellberg J, and Estes M: Updates to the Carbon Bond Mechanism for Version 6 (CB6), in: 9th Annual CMAS Conference, Chapel Hill, NC, 11–13 October 2010, 1–4, https://www.cmascenter.org/conference/2010/abstracts/emery_updates_carbon_2010.pdf (last access: 4 January 2023), 2010.

[R122] Yienger JJ and Levy H II: Empirical model of global soil-biogenic NO*χ*. emissions, J. Geophys. Res.-Atmos, 100, 11447–11464, 10.1029/95JD00370, 1995.

[R123] Young PJ, Archibald AT, Bowman KW, Lamarque J-F, Naik V, Stevenson DS, Tilmes S, Voulgarakis A, Wild O, Bergmann D, Cameron-Smith P, Cionni I, Collins WJ, Dalsøren SB, Doherty RM, Eyring V, Faluvegi G, Horowitz LW, Josse B, Lee YH, MacKenzie IA, Nagashima T, Plummer DA, Righi M, Rumbold ST, Skeie RB, Shindell DT, Strode SA, Sudo K, Szopa S, and Zeng G: Pre-industrial to end 21st century projections of tropospheric ozone from the Atmospheric Chemistry and Climate Model Intercom-parison Project (ACCMIP), Atmos. Chem. Phys, 13, 2063–2090, 10.5194/acp-13-2063-2013, 2013.

[R124] Zaveri RA, Berkowitz CM, Kleinman LI, Springston SR, Doskey PV, Lonneman WA, and Spicer CW: Ozone production efficiency and NO_*X*_ depletion in an urban plume: Interpretation of field observations and implications for evaluating O_3_-NO_*X*_-VOC sensitivity, J. Geophys. Res.-Atmos, 108, 4436, 10.1029/2002JD003144, 2003.

[R125] Zhang R, Cohan A, Biazar AP, and Cohan DS: Source apportionment of biogenic contributions to ozone formation over the United States, Atmos. Environ, 164, 8–19, 10.1016/j.atmosenv.2017.05.044, 2017.

[R126] Zhang S, Zhang Z, Li Y, Du X, Qu L, Tang W, Xu J, and Meng F: Formation processes and source contributions of ground-level ozone in urban and suburban Beijing using the WRF-CMAQ modelling system, J. Environ. Sci, 127, 753–766, 10.1016/j.jes.2022.06.016, 2023. 36522103

[R127] Zhang T, Xu X, and Su Y: Impacts of Regional Transport and Meteorology on Ground-Level Ozone in Windsor, Canada, Atmosphere, 11, 1111, 10.3390/atmos11101111, 2020.

[R128] Zhang Y, Yu S, Chen X, Li Z, Li M, Song Z, Liu W, Li P, Zhang X, Lichtfouse E, and Rosenfeld D: Local production, downward and regional transport aggravated surface ozone pollution during the historical orange-alert large-scale ozone episode in eastern China, Environ. Chem. Lett, 20, 1577–1588, 10.1007/s10311-022-01421-0, 2022.

[R129] Ziemke JR, Oman LD, Strode SA, Douglass AR, Olsen MA, McPeters RD, Bhartia PK, Froidevaux L, Labow GJ, Witte JC, Thompson AM, Haffner DP, Kramarova NA, Frith SM, Huang L-K, Jaross GR, Seftor CJ, Deland MT, and Taylor SL: Trends in global tropospheric ozone inferred from a composite record of TOMS/OMI/MLS/OMPS satellite measurements and the MERRA-2 GMI simulation, Atmos. Chem. Phys, 19, 3257–3269, 10.5194/acp-19-3257-2019, 2019.

[R130] Zohdirad H, Jiang J, Aksoyoglu S, Namin MM, Ashrafi K, and Prévôt ASH: Investigating sources of surface ozone in central Europe during the hot summer in 2018: High temperatures, but not so high ozone, Atmos. Environ, 279, 119099, 10.1016/j.atmosenv.2022.119099, 2022.

