# Supplementary material for: Summertime tropospheric ozone source apportionment study in the Madrid region (Spain)
Source: Atmos Chem Phys. Author manuscript; Available in PMC 2024 Oct 6. (PMC11151812; doi:10.5194/acp-24-4949-2024)
Supplement: SI [file NIHMS1940717-supplement-SI.pdf]

Supplement of Atmos. Chem. Phys., 24, 4949–4972, 2024
https://doi.org/10.5194/acp-24-4949-2024-supplement
© Author(s) 2024. CC BY 4.0 License.

Supplement of

Summertime tropospheric ozone source apportionment study in the Madrid region (Spain)

David de la Paz et al.

Correspondence to: Rafael Borge (rafael.borge@upm.es)

The copyright of individual parts of the supplement might differ from the article licence.

Table S1. WRF model physics options and parametrizations.

Option	Setup
Initialization	GFS
Shortwave radiation	Dudhia scheme
Longwave radiation	GFDL
Land-surface model	Noah LSM
Microphysics scheme	WSM 6-class Graupel scheme
PBL Scheme	YSU scheme
Surface Layer option	Monin-Obukhov
Cumulus Parametrization	No
Nudging	Yes

The WRF model was initialized from global reanalysis made available by NCEP (National Centers for Environmental Prediction) from outputs of the GFS (Global Forecast System) (ds083.0). They have a spatial resolution of 1º x 1º and a temporal resolution of 6 hours ((00Z, 06Z, 12Z, 18Z). Data assimilation was applied (via nudging excluding the planetary boundary layer) for a more realistic representation of meteorological fields using both, surface observations from NCEP ADP Global Surface Observational Weather Data (ds461.0) and vertical soundings from NCEP ADP Global Upper Air Observational Weather Data (ds351.0).

References:

- National Centers for Environmental Prediction/National Weather Service/NOAA/U.S. Department of Commerce. 2004, updated daily. NCEP ADP Global Surface Observational Weather Data, October 1999 - continuing. Research Data Archive at the National Center for Atmospheric Research, Computational and Information Systems Laboratory. https://doi.org/10.5065/4F4P-E398. Accessed 27 January 2016

- National Centers for Environmental Prediction/National Weather Service/NOAA/U.S. Department of Commerce. 2004, updated daily. NCEP ADP Global Surface Observational Weather Data, October 1999 - continuing. Research Data Archive at the National Center for Atmospheric Research, Computational and Information Systems Laboratory. https://doi.org/10.5065/4F4P-E398. Accessed 27 January 2016

- Satellite Services Division/Office of Satellite Data Processing and Distribution/NESDIS/NOAA/U.S. Department of Commerce, and National Centers for Environmental Prediction/National Weather Service/NOAA/U.S. Department of Commerce. 2004, updated daily. NCEP ADP Global Upper Air Observational Weather Data, October 1999 - continuing. Research Data Archive at the National Center for Atmospheric Research, Computational and Information Systems Laboratory. https://doi.org/10.5065/39C5-Z211. Accessed 25 January 2016.

Table S2. Horizontal dimensions and resolution of WRF and CMAQ modeling domains.

Domains	Geographic area	WRF X-Y dimensions (grid cells)	CMAQ X-Y dimensions (grid cells)	Horizontal resolution (km)
D1	Europe	560 x 496	459 x 406	12
D2	Iberian Peninsula	384 x 312	300 x 240	4
D3	Greater Madrid area	256 x 256	136 x 144	1

Table S3. Model performance statistics (dimensionless unless noted otherwise) by station for ground-level O_3 concentration.

STATION	TYPE	FAC2	MB (μgm^{-3})	MGE (μgm^{-3})	NMB	NMGE	RMSE (μgm^{-3})	r	IOA
Arganda del Rey	Industrial	0.96	5.8	14.6	0.07	0.17	18.6	0.84	0.72
Fuenlabrada	Industrial	0.95	9.8	14.4	0.13	0.19	18.7	0.84	0.69
Villa del Prado	Rural	0.99	-0.8	11.8	-0.01	0.13	15.3	0.82	0.72
S.Mde Valdeiglesias	Rural	1.00	0.0	10.5	0.00	0.11	13.7	0.80	0.71
Orusco de Tajuña	Rural	1.00	-10.0	12.7	-0.10	0.12	16.1	0.84	0.66
Guadalix de la sierra	Rural	0.92	7.6	17.9	0.09	0.21	22.7	0.79	0.67
El Atazar	Rural	0.99	-11.2	16.1	-0.11	0.15	20.7	0.69	0.58
Algete	Suburban	1.00	-4.4	13.1	-0.05	0.14	17.1	0.81	0.72
La Sagra	Suburban	0.94	7.3	14.5	0.09	0.18	20.0	0.81	0.71
Mostoles	Suburban	0.94	8.0	15.5	0.10	0.19	20.6	0.83	0.71
Majadahonda	Suburban	0.96	-2.7	15.7	-0.03	0.17	21.4	0.81	0.71
Valdemoro	Suburban	0.91	6.7	16.0	0.08	0.19	22.1	0.80	0.71
Rivas Vaciamadrid	Suburban	0.91	7.0	17.3	0.09	0.21	23.0	0.81	0.70
Torrejon de Ardoz	Suburban	0.90	10.1	17.6	0.13	0.22	23.7	0.82	0.70
Azuqu. de Henares	Suburban	0.95	3.6	16.8	0.04	0.20	21.6	0.78	0.70
Toledo2	Suburban	0.95	-0.9	16.3	-0.01	0.18	22.0	0.72	0.68
Aranjuez	Suburban	0.91	9.3	16.7	0.11	0.20	22.5	0.77	0.67
El Pardo	Suburban	0.92	-0.2	22.2	0.00	0.24	28.0	0.74	0.65
Casa de campo	Suburban	0.94	1.7	20.1	0.02	0.23	26.7	0.61	0.63
Juan Carlos I	Suburban	0.90	-4.6	24.2	-0.05	0.27	31.0	0.61	0.63
Alcorcón	Urb.Background	0.96	4.8	14.6	0.06	0.18	19.7	0.83	0.73
Guadalajara	Urb.Background	0.94	7.4	15.6	0.09	0.19	21.4	0.77	0.70
Tres olivos	Urb.Background	0.92	-4.0	22.8	-0.04	0.25	29.2	0.66	0.63
Villaverde	Urb.Background	0.86	13.1	22.2	0.17	0.29	29.4	0.66	0.61
Farolillo	Urb.Background	0.88	5.8	22.4	0.07	0.27	29.7	0.62	0.62
Retiro	Urb.Background	0.86	11.9	23.0	0.16	0.31	29.3	0.64	0.60
Barajas pueblo	Urb.Background	0.81	11.2	25.1	0.15	0.33	32.2	0.65	0.62
Arturo Soria	Urb.Background	0.84	15.4	23.2	0.22	0.32	29.8	0.63	0.57
Ench de Vallecas	Urb.Background	0.88	6.2	21.1	0.07	0.25	27.9	0.66	0.64
Plaza del Carmen	Urb.Background	0.72	23.9	29.9	0.39	0.48	37.0	0.59	0.47
Segovia 2	Traffic	0.97	3.5	13.6	0.04	0.16	17.0	0.84	0.71
Vill.de Salvanés	Traffic	0.99	3.1	10.6	0.04	0.12	14.6	0.78	0.71
Colmenar Viejo	Traffic	0.99	-0.7	13.0	-0.01	0.14	17.3	0.78	0.69
Alcobendas	Traffic	0.93	-0.8	17.8	-0.01	0.20	23.6	0.80	0.70
Getafe	Traffic	0.92	8.8	16.8	0.11	0.21	23.1	0.80	0.70
Alcala de Henares	Traffic	0.87	10.0	19.3	0.13	0.24	25.0	0.83	0.69
Leganes	Traffic	0.87	12.4	18.4	0.16	0.24	25.7	0.79	0.67
Barrio del Pilar	Traffic	0.88	9.0	20.8	0.11	0.27	28.2	0.64	0.62
Coslada	Traffic	0.79	18.9	24.7	0.27	0.35	31.2	0.80	0.61
Collado Villalba	Traffic	0.78	19.3	23.6	0.26	0.32	31.7	0.73	0.59
Escuelas Aguirre	Traffic	0.83	16.5	23.7	0.24	0.35	29.9	0.63	0.54
Pzs. Fedz Ladreda	Traffic	0.80	22.1	26.7	0.34	0.41	33.4	0.6	0.5

Table S4. Model performance statistics (dimensionless unless noted otherwise) by station type and circulation pattern for ground-level O_3 concentration.

Station	Pattern	n	FAC2	MB (μgm^{-3})	MGE (μgm^{-3})	NMB	NMGE	RMSE (μgm^{-3})	r	IOA
Rural	Accumulation	240	0.98	-6.7	15.29	-0.06	0.14	18.83	0.83	0.66
	Advective	232	0.98	3.1	9.31	0.04	0.11	12.97	0.83	0.73
	Other	3211	0.98	-3.0	14.01	-0.03	0.15	18.30	0.75	0.67
Suburban	Accumulation	474	0.96	-4.8	20.24	-0.05	0.20	26.69	0.76	0.68
	Advective	468	0.92	7.3	13.59	0.10	0.19	19.69	0.75	0.68
	Other	6412	0.94	2.6	17.18	0.03	0.20	23.22	0.73	0.68
Urban background	Accumulation	669	0.89	2.4	23.46	0.03	0.26	31.04	0.69	0.66
	Advective	670	0.89	11.4	16.95	0.17	0.25	22.34	0.72	0.60
	Other	9014	0.89	8.5	20.41	0.11	0.25	27.08	0.68	0.65
Industrial	Accumulation	96	0.95	4.7	16.40	0.05	0.18	20.15	0.86	0.73
	Advective	96	0.97	9.1	12.55	0.13	0.18	15.26	0.82	0.65
	Other	1278	0.95	7.9	14.54	0.10	0.18	18.79	0.83	0.71
Urban traffic	Accumulation	510	0.91	3.5	20.09	0.04	0.22	25.81	0.79	0.69
	Advective	522	0.87	15.8	18.22	0.25	0.28	24.55	0.69	0.55
	Other	7086	0.87	11.0	19.98	0.14	0.25	26.72	0.73	0.65

Table S5. Model (WRF) performance statistics by circulation pattern for basic meteorological variables

Variable	Pattern	FAC2	MB	MGE	NMB	NMGE	r	IOA
**Temperature (T2)**	Accumulation	1.00	-1.4 K	2.0 K	-0.05	0.07	0.92	0.81
	Advection	1.00	-0.5 K	1.5 K	-0.02	0.06	0.96	0.86
	Other	1.00	-0.8 K	1.6 K	-0.03	0.06	0.96	0.85
**Wind speed (WS10)**	Accumulation	0.63	0.9 m/s	1.7 m/s	0.31	0.63	0.30	0.33
	Advection	0.78	0.7 m/s	1.5 m/s	0.17	0.37	0.59	0.55
	Other	0.71	0.5 m/s	1.3 m/s	0.18	0.46	0.58	0.55
**Wind direction**	Accumulation	0.61	-34.3 °	90.7 °	-0.24	0.63	0.26	0.55
	Advection	0.87	6.5 °	34.5 °	0.05	0.25	0.79	0.81
	Other	0.77	-9.2 °	60.8 °	-0.06	0.38	0.53	0.68

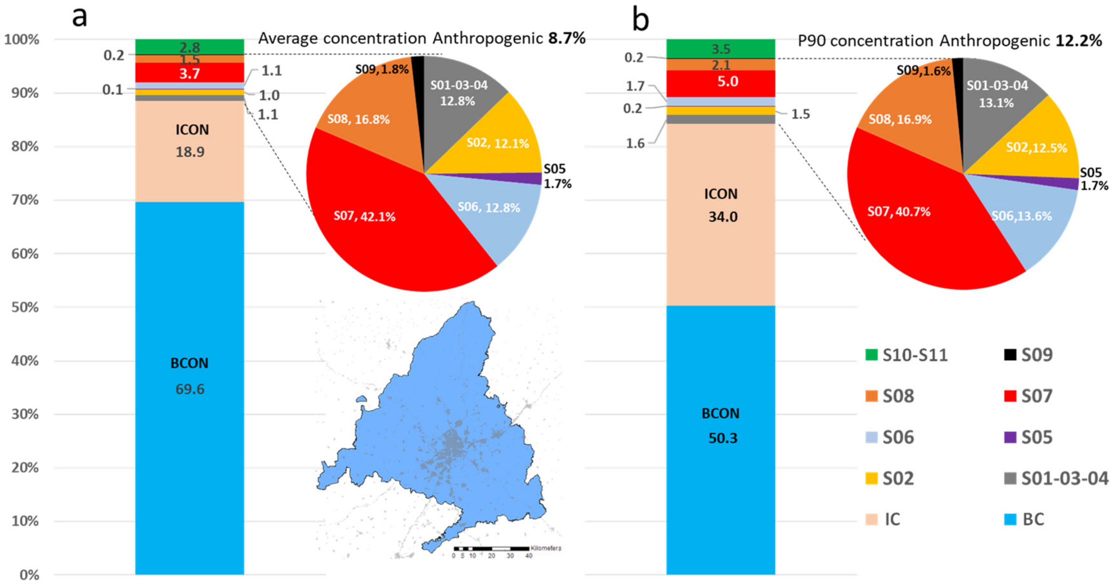

Figure S1. Spatially-averaged source apportionment (%) over the whole Madrid Region for (a) O_3 monthly mean and (b) 90th 1-hour percentile, including the sectoral breakdown within anthropogenic contributions.

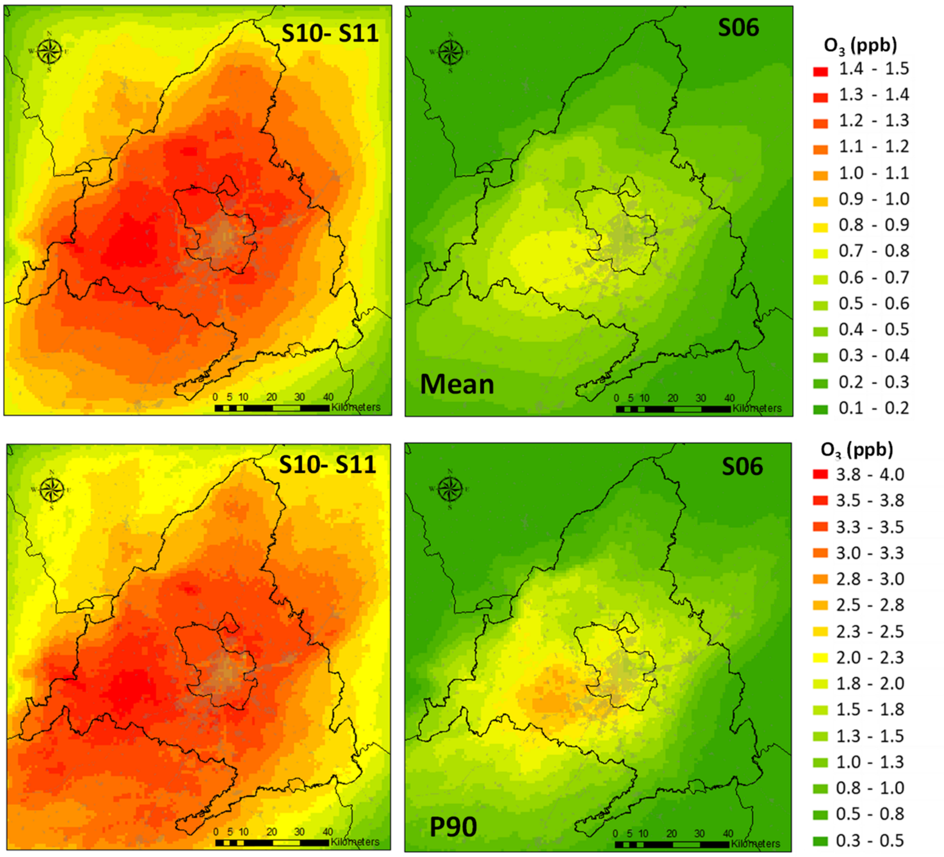

Figure S2. Absolute contribution (ppb) to the monthly mean 1-hour 90th O_3 percentile of the SNAP 06 sector (use of solvents and other products) and SNAP 10 and SNAP 11 (agriculture and nature) emissions.

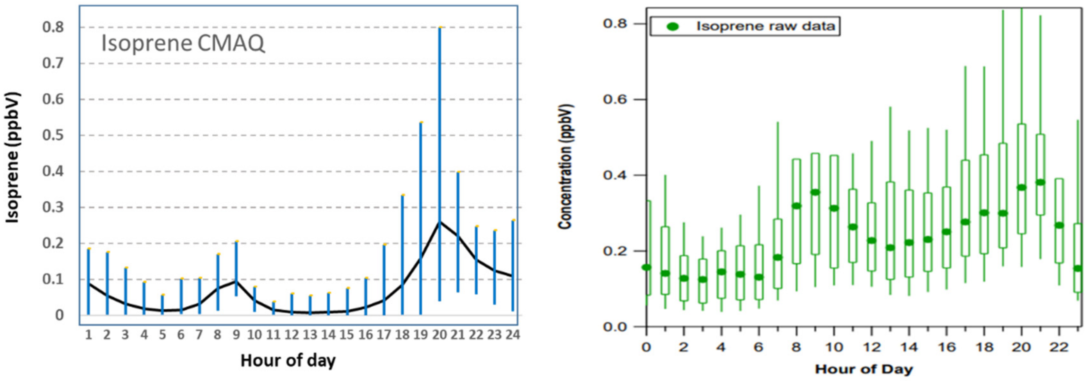

Figure S3. Comparison of isoprene ground-level mixing ratios predicted by CMAQ (left) and measurements made in Majadahonda (suburban site) by Querol et al., (2018) (right). Both graphs present the hourly values during the day averaged over the period July 5th and July 19th. The source of the right-hand panel is Pérez et al., (2016).

Reference:

- Pérez, N., A. Alastuey, C. Reche, M. Ealo, G. Titos, A. Ripoll, M.C. Minguillón, F. J. Gómez-Moreno, E. Alonso-Blanco, E. Coz, E. Díaz, B. Artíñano, S. García dos Santos, R. Fernández-Patier, A. Saiz-López, F. Serranía, M. Anguas-Ballesteros, B. TemimeRoussel, N. Marchand, D. C. S. Beddows, R. M. Harrison y X. Querol. Campaña intensiva de medidas de UFP, O3 y sus precursores en el área de Madrid: medidas en superficie., https://www.miteco.gob.es/content/dam/miteco/es/calidad-y-evaluacion-ambiental/temas/atmosfera-y-calidad-del-aire/anexo_informea33_madrid_tcm30-561368.pdf (last access: [January 22, 2024]), 2016.

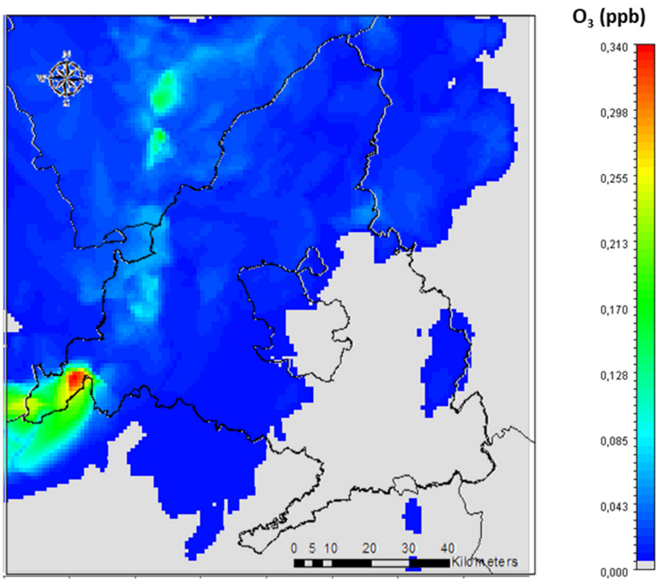

Figure S4. Maximum 1-hour attribution of stratospheric transport (ST) to ground-level O_3.

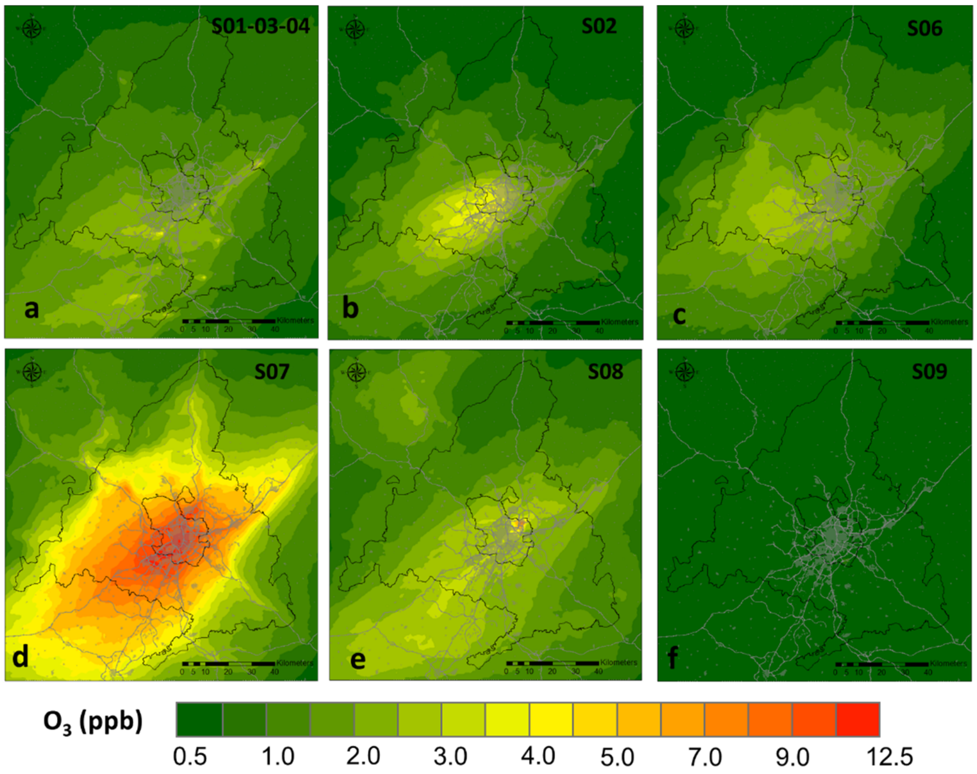

Figure S5. Absolute contribution to the 1-hour 90th O_3 percentile of the main emitting sectors.

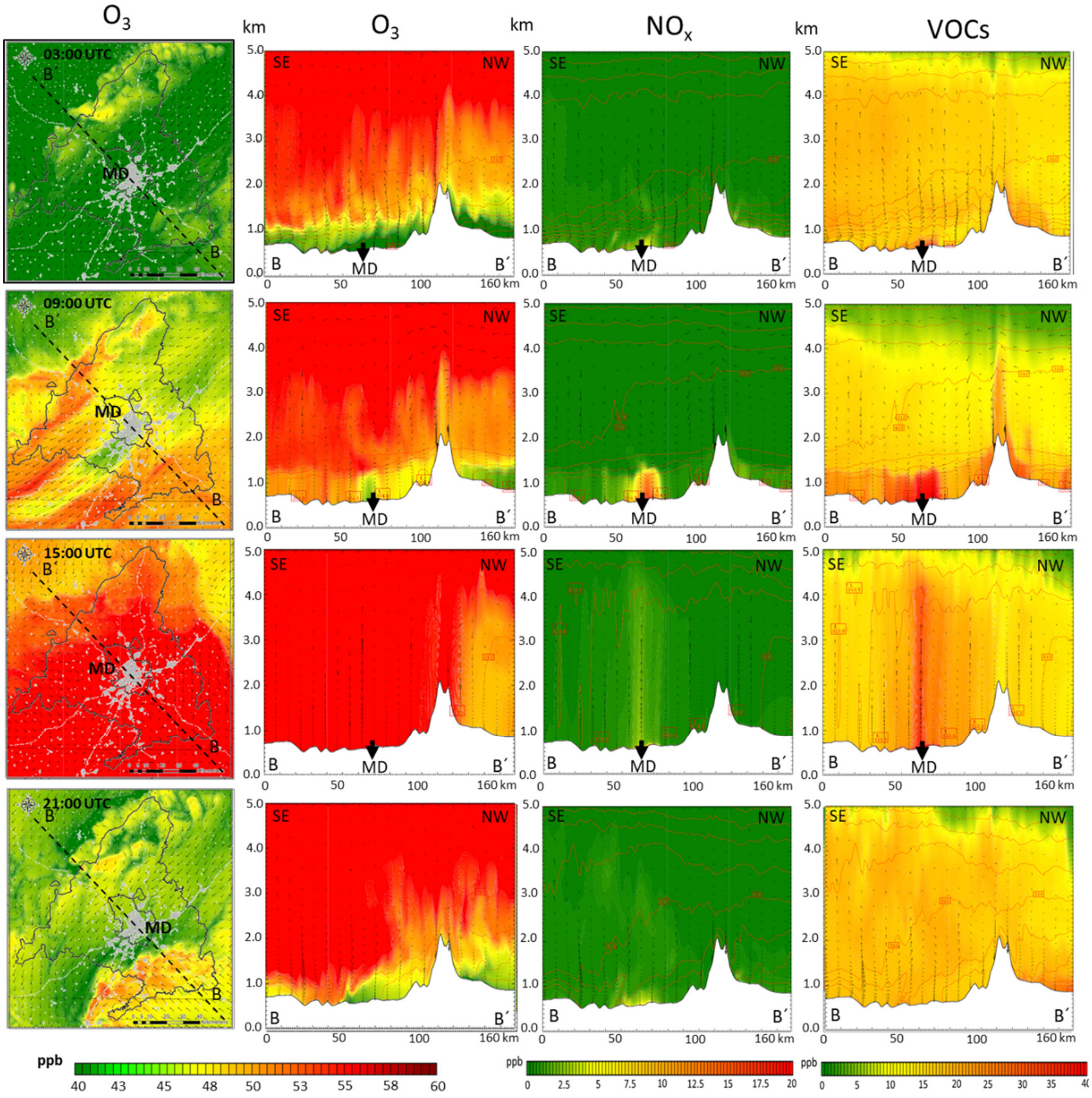

Figure S6. Accumulation period: evolution during July 27th. From left to right, plan view and SE-NW cross section (up to 5 km height) O_3 mixing ratios (ppb), NOx (ppb) and VOCs (ppb) at 3:00, 9:00; 15:00, 21: 00 UTC hours. MD = Madrid City.

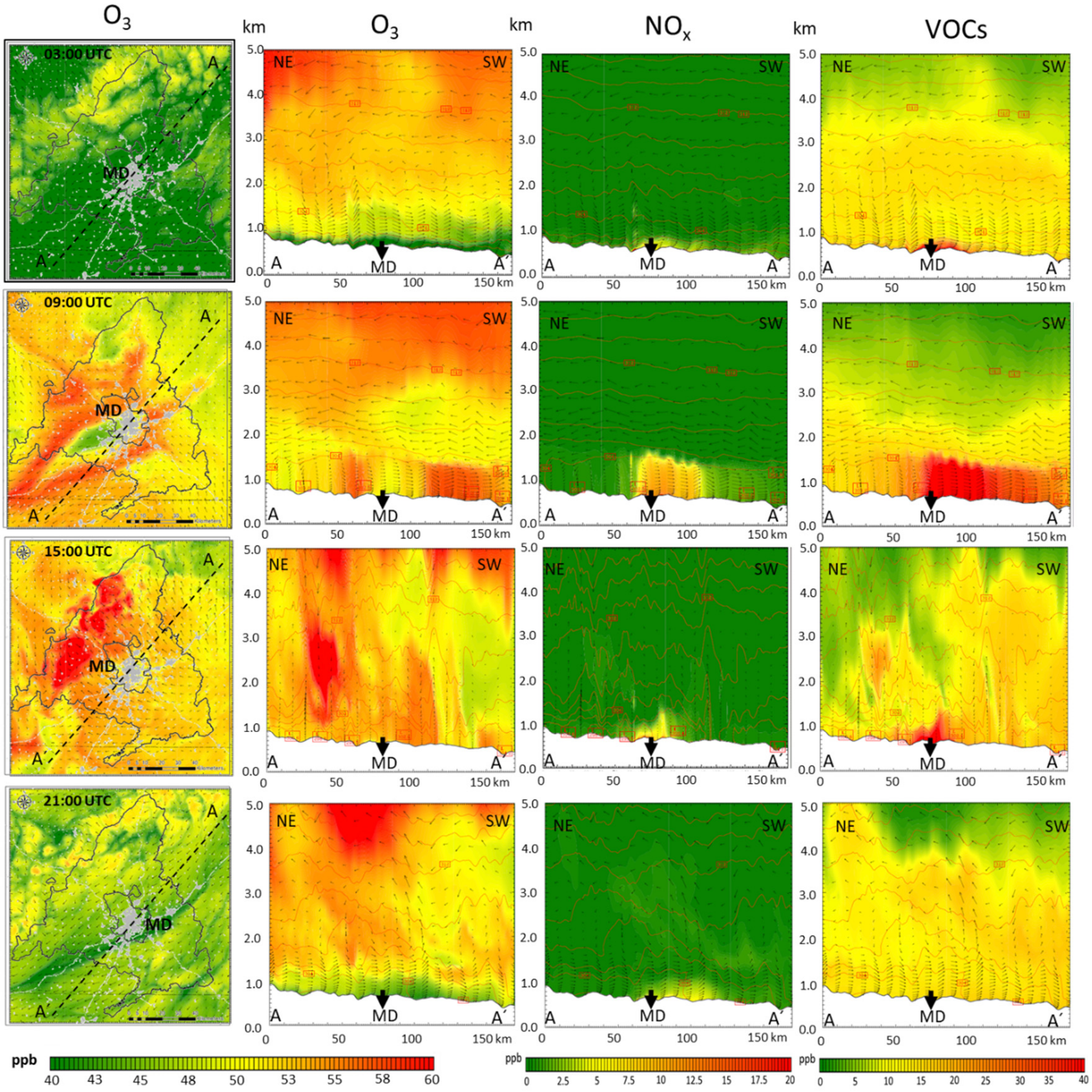

Figure S7. Accumulation period: evolution during July 6th. From left to right, plan view and NE-SW cross section (up to 5 km height) O_3 mixing ratios (ppb), NOx (ppb) and VOCs (ppb) at 3:00, 9:00; 15:00, 21: 00 UTC hours. MD = Madrid City.

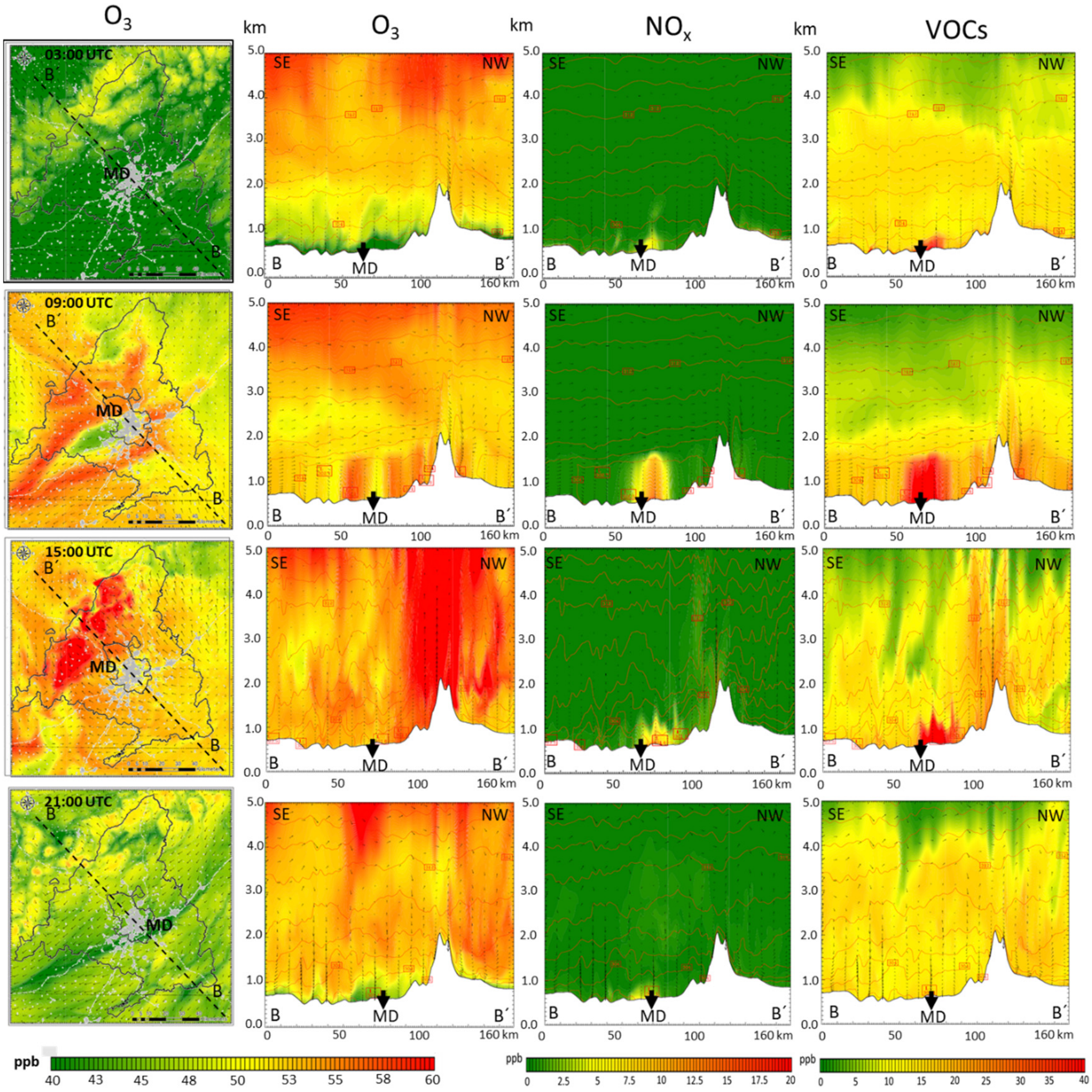

Figure S8. Accumulation period: evolution during July 6th. From left to right, plan view and SE-NW cross section (up to 5 km height) O_3 mixing ratios (ppb), NOx (ppb) and VOCs (ppb) at 3:00, 9:00; 15:00, 21: 00 UTC hours. MD = Madrid City.

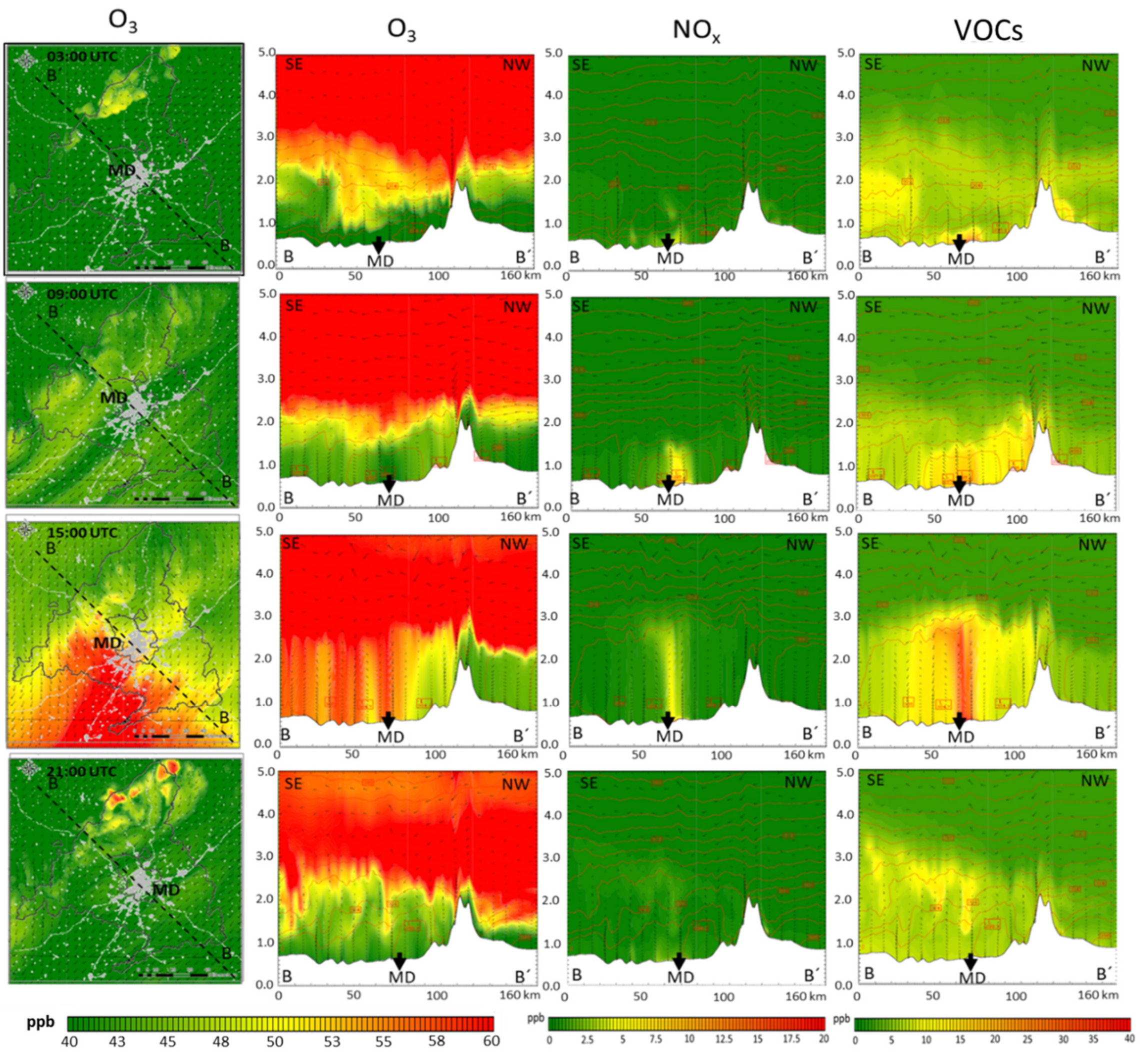

Figure S9. Advection period: evolution during July 13th. From left to right, plan view and SE-NW cross section (up to 5 km height) O_3 mixing ratios (ppb), NOx (ppb) and VOCs (ppb) at 3:00, 9:00; 15:00, 21: 00 UTC hours. MD = Madrid City.

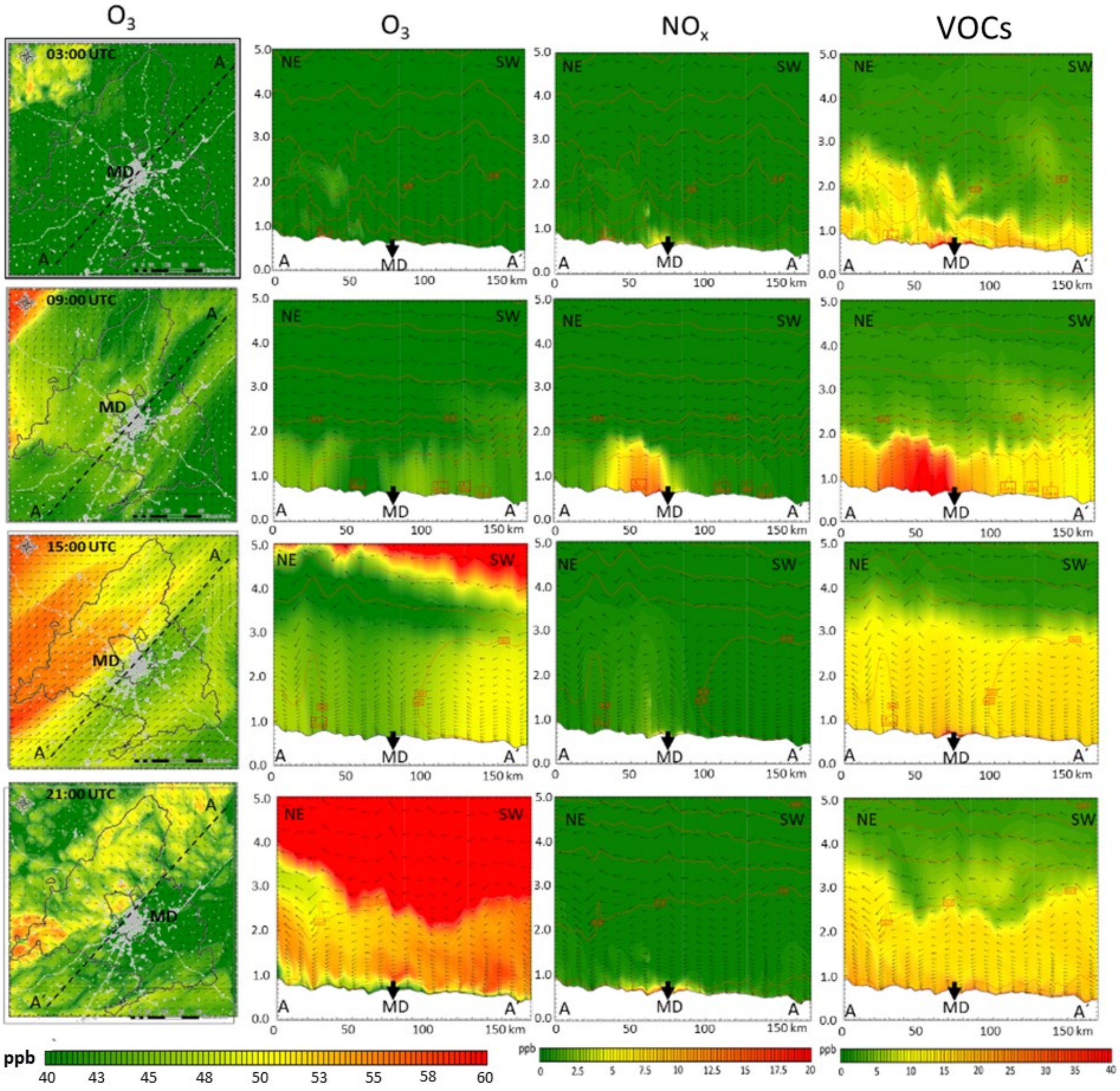

Figure S10. Advection period: evolution during July 20th. From left to right, plan view and NE-SW cross section (up to 5 km height) O_3 mixing ratios (ppb), NOx (ppb) and VOCs (ppb) at 3:00, 9:00; 15:00, 21: 00 UTC hours. MD = Madrid City.

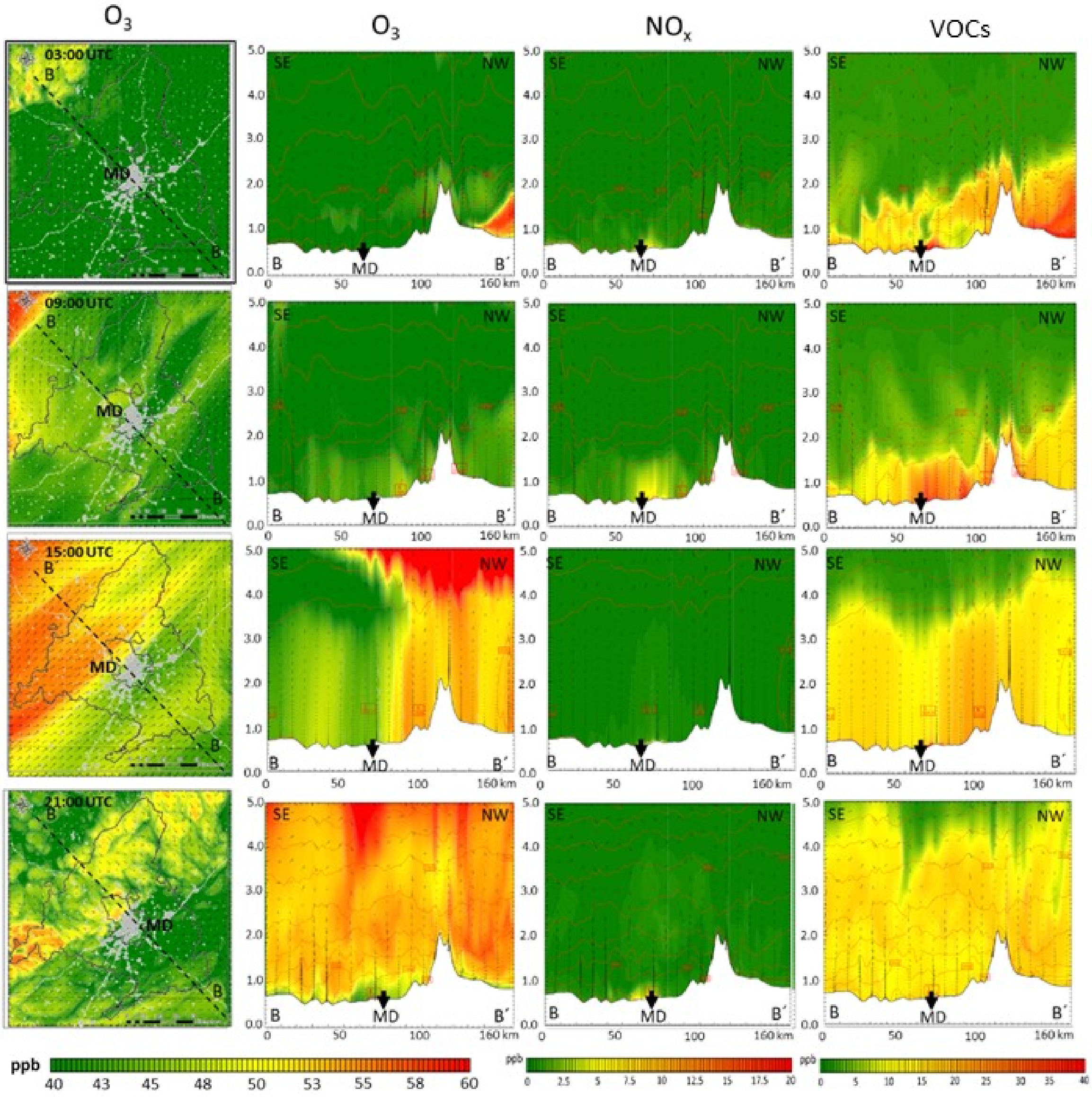

Figure S11. Advection period: evolution during July 20th. From left to right, plan view and SE-NW cross section (up to 5 km height) O_3 mixing ratios (ppb), NOx (ppb) and VOCs (ppb) at 3:00, 9:00; 15:00, 21: 00 UTC hours. MD = Madrid City.

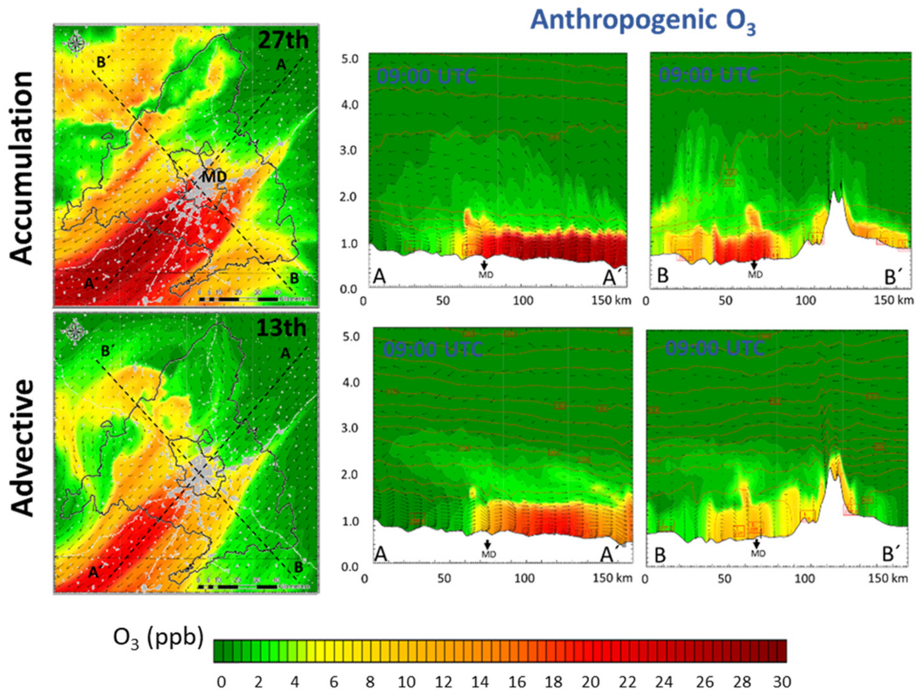

Figure S12. O_3 mixing ratios (ppb) at 09:00 UTC for July 27th (accumulation period) and July 13th (advective period). From left to right, plan view, NE-SW and SE-NW cross sections (up to 5 km height). MD = Madrid City.

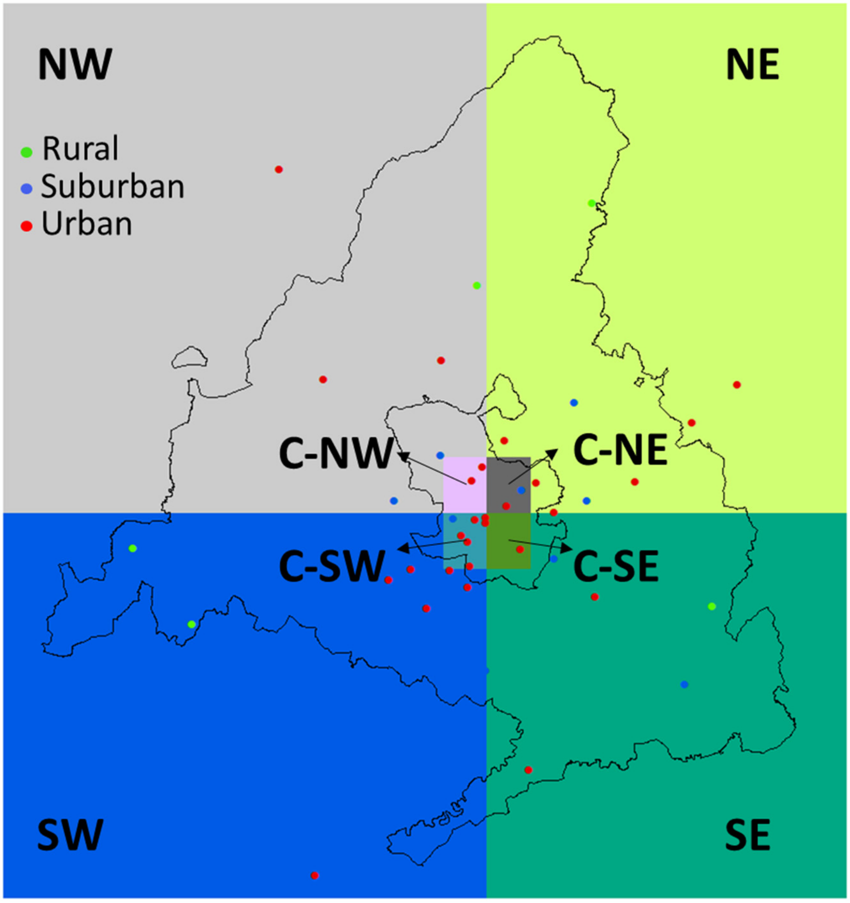

Figure S13. Geographical division (quadrants) of the study area for the analysis of individual monitoring station locations.

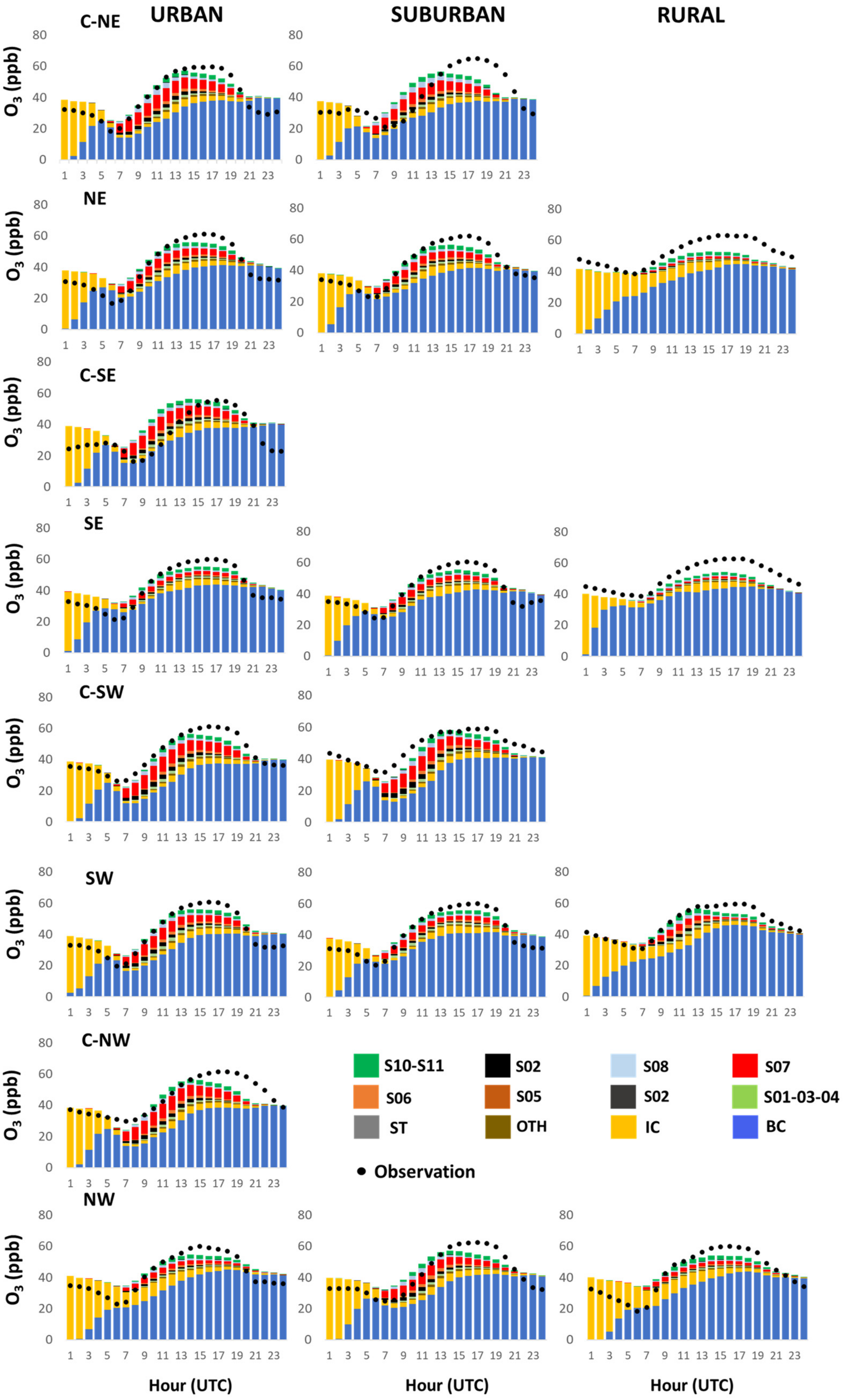

Figure S14. Hourly contribution to ground-level O_3 (ppb) for the monthly average at the location of monitoring sites by geographical quadrant.

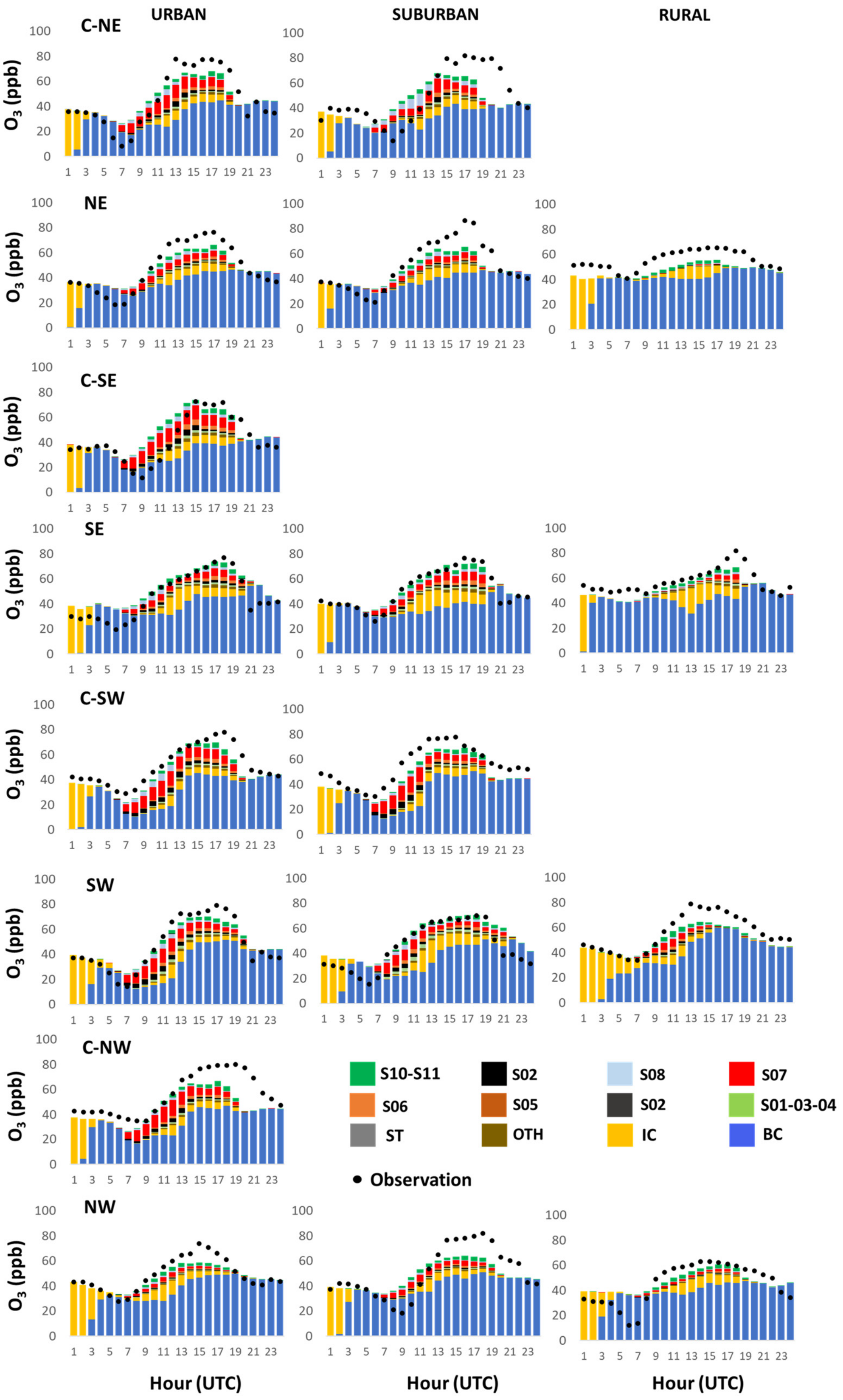

Figure S15. Hourly contribution to ground-level O_3 (ppb) for July 27th, 2016 at the location of monitoring sites by geographical quadrant.

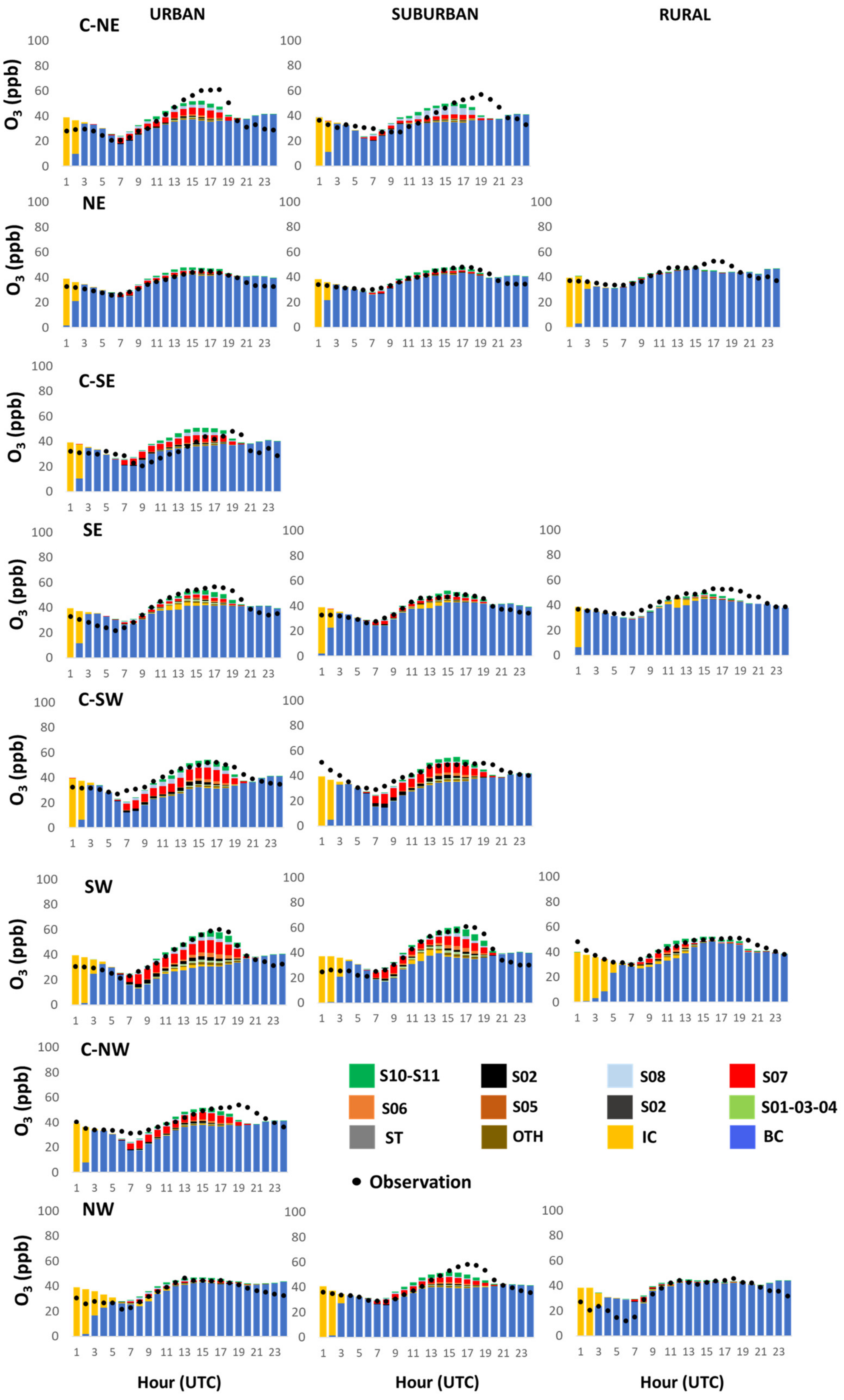

Figure S16. Hourly contribution to ground-level O_3 (ppb) for July 13th, 2016 at the location of monitoring sites by geographical quadrant.
